# Revisiting Temozolomide's role in solid tumors: Old is gold?

**DOI:** 10.7150/jca.94109

**Published:** 2024-04-22

**Authors:** Dimitrios Matthaios, Ioanna Balgkouranidou, Konstantinos Neanidis, Andreas Sofis, Anastasia Pikouli, Konstantinos Romanidis, Aglaia Pappa, Michael Karamouzis, Anna Zygogianni, Charalampos Charalampidis, Paul Zarogoulidis, George Rigas, Alex Galanis

**Affiliations:** 1 Department of Oncology, General Hospital of Rhodes, Rhodes, Greece.; 2 Department of Oncology, Democritus University of Thrace, Alexandroupolis, Greece.; 3 Oncology Department, 424 General Military Training Hospital, Thessaloniki, Greece.; 4 Attikon University Hospital, Athens, Greece.; 5 Third Department of Surgery, Attikon University Hospital, Athens, Greece.; 6 Second Department of Surgery, University General Hospital of Alexandroupolis, Democritus University of Thrace Medical School, Alexandroupolis, Greece.; 7 Department of Molecular Biology & Genetics, Democritus University of Thrace, Alexandroupolis, Greece.; 8 Molecular Oncology Unit, Department of Biological Chemistry, Medical School, National and Kapodistrian University of Athens, Athens, Greece.; 9 Radiation Oncology Unit, 1st Department of Radiology, Aretaieion University Hospital, School of Medicine, National and Kapodistrian University of Athens, Athens, Greece.; 10 Surgery Department, ``Genesis`` Private Hospital, Thessaloniki, Greece.; 11 Pathology Department, University of Cyprus, Cyprus.; 12 Pulmonary-Oncology Department, General Clinic Euromedice, Thessaloniki, Greece.; 13 Oncology Department, Private General Clinic of Volos, Volos, Greece.

**Keywords:** temozolomide, solid tumors

## Abstract

Temozolomide is an imidazotetrazine with a long history in oncology especially for the high grade malignant glioma and metastatic melanoma. However, last year's new indications for its use are added. Its optimum pharmacodynamic profile, its ability to penetrate the blood-brain barrier, the existence of methylation of MGMT in solid tumors which enhances its efficacy, the identification of new agents that can overcome temozolomide's resistance, the promising role of temozolomide in turning immune cold tumors to hot ones, are leading to expand its use in other solid tumors, giving oncologists an additional tool for the treatment of advanced and aggressive neoplasms.

## 1. Introduction: General principles of action

The chemical formula for temozolomide, also known as CCRG 81045, M & b 39831, SCH 52365, and NSC 362856, is 8-carbamoyl-3-methylimidazo-[5,1-d]-1,2,3,5-tetrazin4(3H)1. The U.S. Food and Drug Administration approved temozolomide (Scheder Corporation's Temodar® capsules) on March 15, 2005, for the concurrent treatment of adult patients with newly diagnosed glioblastoma multiforme (GBM) with radiation therapy and later as a maintenance treatment. In 1999, temozolomide (TMZ) was granted expedited approval for the management of adult patients suffering from refractory anaplastic astrocytoma. The NCCN recommendations also recommend temozolomide for the treatment of advanced or metastatic melanoma (1). The NCCN guidelines state that temozolomide-based therapy is also recommended for the management of advanced pancreatic neuroendocrine tumors. Soft tissue sarcomas with non-specific histologies can also be treated with TMZ as an active single agent. Furthermore, temozolomide activity combined with irinotecan is recognized by NCCN recommendations as a second-line therapy for Ewing's sarcomas (relapsed/refractory or metastatic disease). Temozolomide is a prodrug that breaks down spontaneously into the reactive intermediate 5-(3-methyl-1-triazeno)imidazole-4-carboxamide (MTIC) in solution at physiological pH. MTIC is an alkylating chemical that methylates adenine at the N3 position as well as guanine at the N7 and O6 positions. A perpetual cycle of DNA base mismatch and repair is brought on by these methyl adducts, culminating in strand breaks and cellular death 2, 3, 4, 5. It is believed that the most common cytotoxic adduct is methylation of guanine at the O6 site 6, 7. The primary lesion caused by MTIC methylation of guanine's O6-position mismatches with thymine in double-stranded DNA (O6G-T) during the first cell cycle following therapy. Recurrent GT-mismatches cause mismatch repair, which is induced by this mismatch and can lead to double strand breaks or a secondary lesion. This secondary lesion is a location created by defective mismatch repair, which halts replication and causes sister chromatid exchange, tertiary lesion formation, or other abnormalities 8-12. Therefore, tumor cells die as a result of tertiary lesions created by improper mismatch repair rather than the main lesions brought on by TMZ. Other methods of action have also been examined and investigated. Two days after treatment, TMZ can cause a prolonged G2-M arrest mediated by p53 and p21WAF1/Cip1, with most cells senescing over ten days, while a small percentage of cells undergo apoptosis. On the other hand, in p53 deficient cells, TMZ causes a transient G2-M arrest along with little alterations in p53 or p21WAF1/Cip1 expression 13. Both on its own and in conjunction with other compounds, TMZ has a number of benefits. These features include the capacity to penetrate the central nervous system, stability in acidic environments that for total oral absorption, and quick and widespread tissue dispersion. Prodrug of 5-(3-methyltriazen-1-yl)imidazole-4-carboximide 5,14, the active species that methylates DNA (7), TMZ is similar to DTIC. Only hepatic p450 metabolism, which is extremely erratic and changeable, can activate DTIC, although TEM's metabolic activation happens spontaneously and fully at physiological pH 5, 7. Furthermore, TMZ is effective against a wide range of conditions, such as melanoma, mycosis fungoides, and recurrent high-grade astrocytomas 5, 15-18. In Mer-human brain tumor xenografts resistant to BCNU (bis-chloroethylnitrosourea), TMZ also exhibits action 19.

In this review we aimed to investigate the uses of temozolomide in solid tumors, excluding melanoma and brain tumors, in which its value is recognized and broadly accepted.

## 2. Search strategy and selection criteria

The references for this review were found by searching PubMed and PMC between 1980 and November 2023 using the keywords "temozolomide," "temodal," and "solid tumors." Reviewing was limited to papers published in English and French. Originality and relevance to the wide scope of this Review were the guiding principles in the creation of the final reference list. Using the same method, Clinicaltrials.gov's ongoing and completed trials were found (Table 1).

## 3. How to measure MGMT function

**Figure 1:**
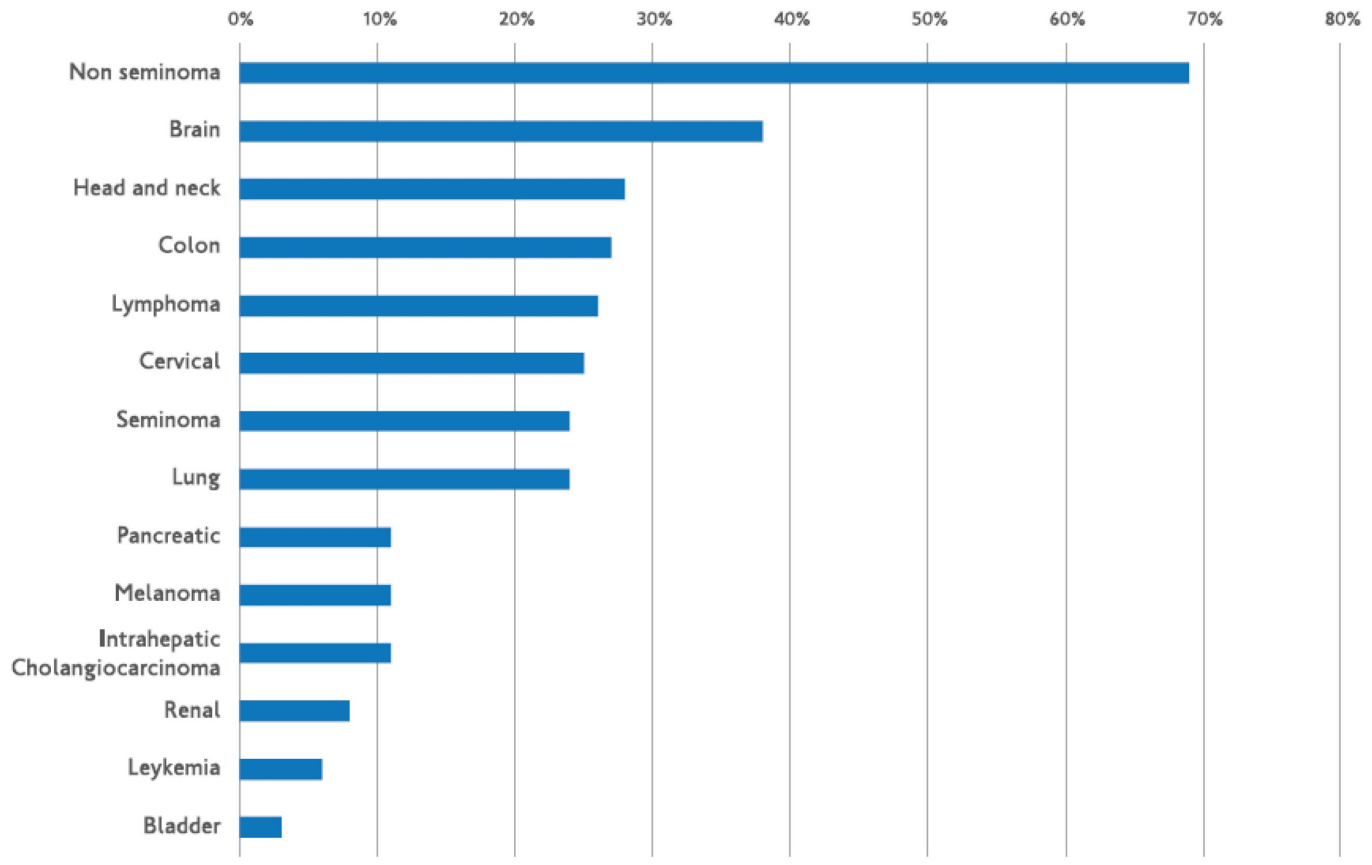
Frequency of MGMT promoter methylation in different solid tumors. Markus Christmann* et al.* Bernd Kaina O(6)-Methylguanine-DNA methyltransferase (MGMT) in normal tissues and tumors: enzyme activity, promoter methylation and immunohistochemistry Biochim Biophys Acta. 2011 Dec;1816(2):179-90.

The methylation-specific polymerase chain reaction test (MS-PCR) is used in most investigations on MGMT (methyl-guanine methyl transferase) promoter methylation 20-22. Using immunohistochemistry to determine the MGMT gene's function is an additional approach 23-25. Nonetheless, there is inconsistent information regarding both MGMT immunoreactivity and MGMT promoter methylation 26-28. If only FFPE tissue is available, immunohistochemistry is a more dependable approach than MS-PCR. Nonetheless, there is disagreement on the significance of MGMT-immunoreactivity, particularly in light of its correlation with the methylation state of the MGMT promoter 26-28,29, 30. Under some circumstances, it has been demonstrated that extensive MGMT promoter methylation correlates with MGMT gene expression 31. However, a negative MGMT-immunostaining did not correspond with a specific promoter methylation status, which may indicate that MGMT protein expression is not always coupled with MGMT promoter methylation. In addition to promoter methylation, gene deletion or mutation can result in a reduction of protein production, which is one of the several methods of gene silencing that have been reported. Furthermore, as MGMT is an inducible protein 29,32,33, a lack of immunoreactivity at the time of diagnosis may not indicate that the protein has the capacity to operate as intended. Such therapy may be expected to be responsive in tumors with low or no MGMT levels because of MGMT being epigenetically silenced by methylation of CpG islands in the promoter region 20. Figure 1 shows the frequency of MGMT promoter methylation in various tumor types. Lesions brought on by chemotherapy do not heal and cause cytotoxicity and apoptosis. Numerous investigations have looked into the relationship between the methylation status of the MGMT promoter and how tumors react to alkylating drugs, such as carmustin, lomustine, and temozolomide 20, 21, 34. After receiving TMZ therapy, patients with methylation MGMT promoters for glioblastoma multiforme fared better than those without such a promoter. This supports the theory that the tumor's vulnerability to alkylating drugs is correlated with MGMT inactivation caused by aberrant promoter methylation 34.

## 4. Progress in methods of detection of MGMT promoter methylation status

While methylation-specific PCR, pyrosequencing, or methylation arrays are recommended for the detection of MGMT promoter methylation, the European Society of Medical Oncology no longer recommends immunohistochemistry, despite the fact that it was once the basis method for MGMT methylation detections 35, 36. On the other hand, pyrosequence can be very informative in evaluating the proportion of MGMT methylation, which can predict the volume response and prognosis of patients with residual GBM 37-39,40. It is recommended to use a biological cutoff of 10% or 21% of the receiver operating characteristic. Other approaches that show promise for MGMT promoter methylation detection include endonuclease-resistant DNA methylation quantification, Lab on Chip compatible isothermal amplification, and two probe quantification of MSB 40-42. In terms of defining the ideal cutoff, research indicates that, for CpG sites 74-78, a cutoff of 9% is preferable to a higher cutoff of 28% or 29% 38. Furthermore, it appears that a PSQ score of 10% for MGMT promoter methylation can classify patients into a "transition zone" or "gray area" since it may increase their susceptibility to TMZ treatment 43. Additionally, advances in radiomics techniques are being made in an effort to provide a noninvasive, preoperative method of MGMT promoter methylation detection 44-49. Ultimately, significant advancements have also been made in the examination of the MGMT methylation status in peripheral blood and cerebral fluid 50-51.

## 5. Resistance to temozolomide

**Figure 2:**
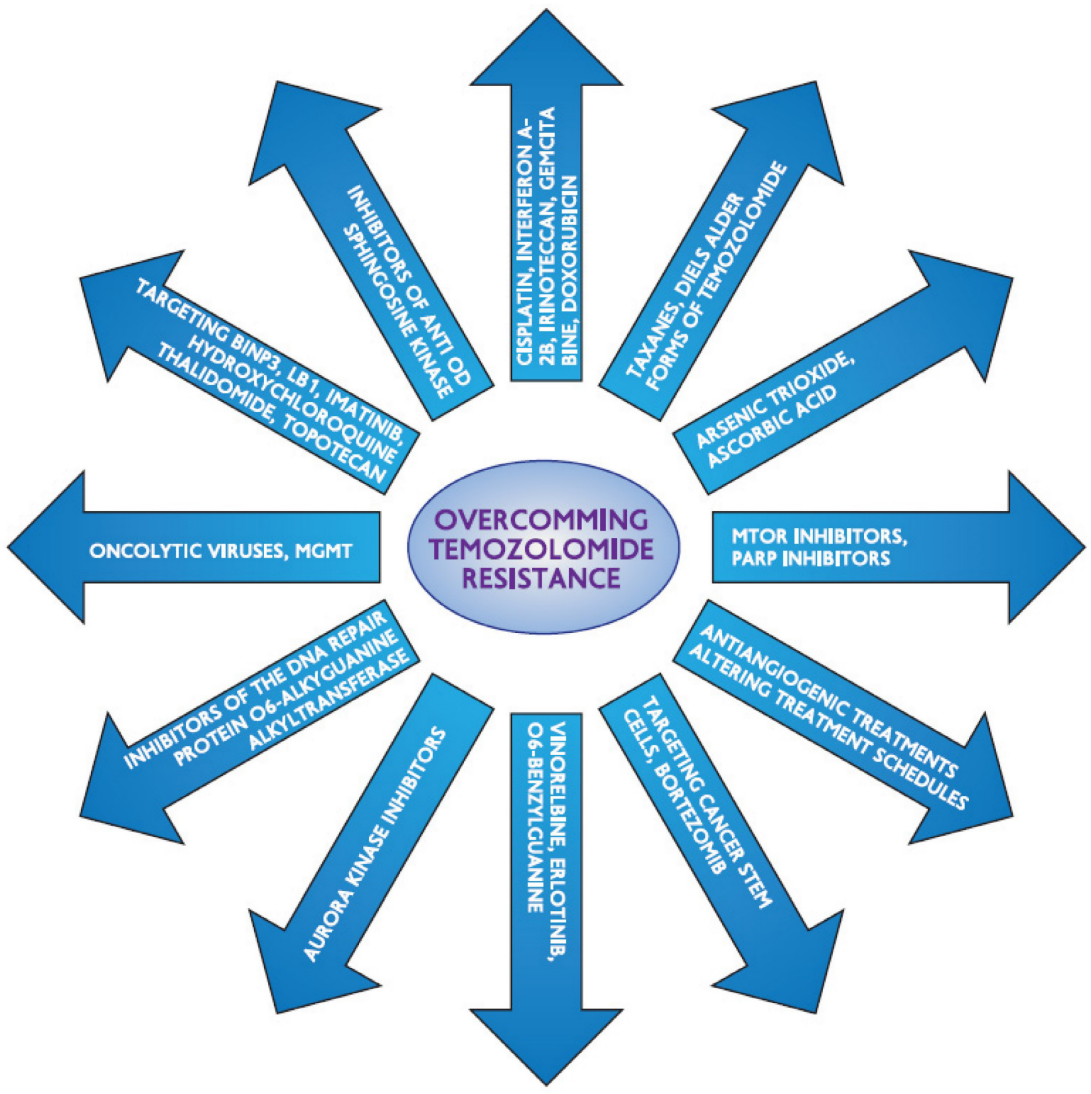
Strategies to overcome temozolomide's resistance.

The DNA repair protein O6-alkylguanine-DNA alkyltransferase (AGT), which eliminates the methyl adduct from the O6 position of guanine, mediates resistance to temozolomide. By acting as a substrate and moving its benzyl group to the active site of AGT, O6-benzylguanine (O6BG), a modifying agent, inactivates and depletes AGT. For TMZ-induced methylating cytotoxicity to occur, an MMR repair mechanism must be functional. A weak MMR pathway will cause the alkylation damage to be tolerated. Melanomas are less common than brain malignancies in this regard. Furthermore, as observed in melanomas with bcl-2 overexpression, tumor cells' capacity to evade apoptosis is another element that may contribute to temozolomide resistance 52. In a study looking at glioblastoma chemoresistance, Beier et al. came to the conclusion that MGMT protein expression is linked to a high level of TMZ resistance in cancer stem cells (CSC). Furthermore, the authors observed that neurosphere-forming cells lacking MGMT expression were vulnerable to TMZ when examined in depth. Additionally, they discovered that inconsistent experimental outcomes could arise from varying TMZ timings and dosages. Additionally, they noted that environmental conditions, such as hypoxia in the glioblastoma's core, could be a component in the CSC's resistance to TMZ. They came to the conclusion that TMZ resistance is impeded by a number of signaling pathways, including those of Shh, IGF-1/PI-3 kinase, NOTCH, and STAT3 53. For TMZ to induce harmful double strand breaks, the mismatch repair system must be functional. Therefore, TMZ resistance was mediated by changes in the main, critical component of the mismatch repair mechanism, mutS homolog 6 (MSH6), particularly in recurrent GBM following TMZ-based radiochemotherapy 54, 55. A fraction of GBM recurrences following radiation therapy and TMZ treatment had inactivated mutations in the mismatch repair gene MSH6, which results in the loss of MSH6 immunostaining. During TMZ treatment, loss of MSH6 was associated with tumor development 55, 56. TMZ resistance was linked to MSH6 inactivation and mutation in GBMs after TMZ therapy, both in vitro and in vivo 55. The double strand breaks that cause cell death are inhibited by a well-established resistance mechanism. Mutations affecting the apoptotic cascade, which carries out double stranded break-induced apoptosis, as well as p53 and Poly(ADP-ribose)polymerase (PARP) signaling, are among the signaling cascades involved 57,58. Glutathione-S-transferase is a protein that contributes to chemoresistance but is not as well understood. Figure 2 shows methods for overcoming the resistance to temozolomide.

## 6. Temozolomide's efficacy in solid tumors other than brain and melanoma

### 6.1. Colorectal cancer

The removal of alkyl groups from guanine's O6-position is carried out by the DNA repair gene O6-methylguanine-DNA methyltransferase (MGMT). If dormant, it could contribute to the initial stages of colorectal cancer by raising the rate of mutations, especially G-to-A point mutations in the KRAS gene 59, 60. The MGMT-encoded protein fixes DNA damage caused by alkylating chemicals in a variety of tumor types 61,62. Hypermethylation of CpG island in MGMT promoter is linked to epigenetic silencing of MGMT during colorectal carcinogenesis 63. Reduced DNA-repair of O6-alkylguanine adducts is caused by this transcriptional gene suppression, which increases chemosensitivity to alkylating drugs, especially dacarbazine and its oral prodrug temozolomide 64. 32 patients with advanced chemorefractory colorectal cancer with MGMT promoter methylation were enrolled in our phase 2 research. In 4-week cycles, the patients received treatment with TMZ at a dose of 150 mg/m2/day for 5 days in a row. The course of treatment was followed until the condition worsened, or at least six cycles. At 12%, the objective response rate reached the pre-established threshold for activity that showed promise. There was a median of 1.8 months for progression-free survival and 8.4 months for overall survival. When compared to patients with any RAS or BRAF mutation, those with KRAS, BRAF, and NRAS wild-type CRC demonstrated a significantly greater response (44% versus 0%; P = 0.004) (65). In order to investigate the effectiveness of TMZ in conjunction with pegylated liposomal doxorubicin for the treatment of brain metastases from different solid tumors, Caraglia et al. carried out a phase 2 trial. A total of 36.8% (95% CI:19.1-59.2) of the 19 patients in the cohort had a complete response (CR), four had a partial response (PR), and three had a complete response (CR). This response rate exceeded the research design's target activity. The primary diagnosis in three cases was colorectal cancer. Of them, two (67%) reported giving only a partial answer 66. The idea of using temozolomide as an induction treatment that sensitizes patients with MSS and MGMT-silenced CRC to later use of immunotherapy was supported by three trials by Gonzalez et al., the MAYA trial, and the Arethusa trial, as we will analyze later in our review. This opened the door for a strategy that turns immune cold tumors into hot ones 67-69.

### 6.2. Neuro-endocrine tumors, melanoma

Temozolomide was prescribed to patients with malignant endocrine tumors because to the comparable mechanisms of action between dacarbazine and TMZ. In mice, TMZ has been shown to be less harmful than dacarbazine 5. There was no significant difference in the safety of the drugs between TMZ and dacarbazine in a randomized phase III research conducted on patients with advanced metastatic melanoma 1. After treatment with oral TMZ, there was a greater systemic exposure (area under the curve) to the parent drug and its active metabolite, 5-(3-methyl-triazeno)imidazole-4-carboximide, compared to dacarbazine administered intravenously. The most frequent toxicities associated with TMZ were mild to severe nausea and vomiting that could be treated with ease, as well as a noncumulative temporary myelosuppression 1. Additionally, patients' health-related quality of life was enhanced with TMZ therapy. Furthermore, it has been demonstrated that TMZ is effective in glioblastoma patients and enhances survival when combined with radiation therapy in this context 70. Interesting outcomes of TMZ in patients with endocrine malignancies were documented in two investigations 71, 72. The effectiveness and safety of TMZ in treating patients with malignant digestive endocrine tumors were evaluated by Mairie et al. TMZ given at doses of 200 mg/m2 daily for 5 days every 28 days resulted in the disease stabilization of 81% of patients in their cohort of 21 patients with metastatic well-differentiated digestive endocrine tumors 73. In a phase II trial, the combination of thalidomide plus TMZ was found to have an overall radiologic response rate of 25% over a median of 13.5 months for the treatment of metastatic neuroendocrine tumors 71. A 2006 American Society of Clinical Oncology meeting abstract featured a retrospective analysis of TMZ and capecitabine combined treatment for pancreatic neuroendocrine tumors. The study found that a median of 9.5 months was experienced by 6% of patients who experienced a full response and 53% who experienced a partial response 74. A phase II study with TMZ plus bevacizumab, presented at the same meeting, revealed an overall response rate of 14% 75. The lack of benefit from this treatment in some NETs, and in carcinoids specifically, may be explained by the dependency of TMZ response on poor MGMT expression. Kulke et al. evaluated 76 patients who were getting temozolomide-based therapy in a retrospective manner. About 33% of patients with pancreatic NETs (11/35 patients) had a radiographic response (determined by RECIST criteria), but 0% of patients with carcinoid tumors (0/38) had a radiographic response (P<0.001). Complete lack of MGMT expression appeared to characterize patients with pancreatic NET (5/8 pancreatic NET and 0/13 carcinoid tumors) who benefit significantly from temozolomide in 21 available specimens 76. Ekeblad et al. looked at 36 patients with advanced neuroendocrine tumors to see if TMZ was effective. Of the patients, 14% had a radiologic response, while 53% had stable illness 72. Hirohata et al. investigated the function of DNA mismatch repair protein (MSH6) as a response biomarker in patients receiving TMZ treatment for pituitary cancer and atypical pituitary adenomas. They discovered a positive correlation between the TMZ response and the immunopositivity of MSH6 77. Based on its method of action and advantageous toxicity profile, the CAPTEM regimen is currently frequently utilized in clinical practice, particularly for G2-G3 NETs. 78,79. Only metastatic or unresactable GEP-NENs G3 with a Ki-67 >20% and <55% treated with CAPTEM were included in a recent single-arm phase II trial. The results indicated a significant improvement in PFS and OS in NETs compared to NEC (9.3 months versus 3.5 months, P = 0.005, not reached versus 6.2 months, P = 0.004). Furthermore, CAPTEM is the recommended course of action for patients with well-differentiated G3 NETs, as evidenced by the decreased ORR (14.3% versus 34.8%, P = 0.393) and DCR (42.9% against 87.0%, P = 0.033) in NEC patients compared to NETs G (31). In 144 patients with advanced low or intermediate grade pNETs, one of the most recent randomized phase II trials (E2211) compared temozolomide monotherapy to CAPTEM, establishing CAPTEM as the standard chemotherapy for advanced pNETs. Despite the absence of a statistically significant difference in ORR (33.3% for CAPTEM vs. 27.8% for TEM, p = 0.47) between the two treatment modalities, the combination was linked to a considerably longer median PFS (22.7 vs. 14.4 months) than TEM monotherapy 81. It is noteworthy that the ORR for NENs treated with CAPTEM was greater than the ORR for the majority of licensed therapies (≈ 30%). The best order of treatment is still up for debate because there hasn't been a prospective, randomized clinical trial contrasting CAPTEM with single-agent tyrosine kinase inhibitors 82-84. In a recent review, Arrivi et al. draw the conclusion that, while pNETs have more robust efficacy data, which has led to the widespread adoption of the CAPTEM regimen in cancers of the pancreas, CAPTEM appears to be a safe and effective treatment for patients with advanced well-moderately differentiated NENs of the gastrointestinal tract, the lung, and those of unknown origin 85.

### 6.3. Breast Cancer

Because temozolomide is an oral medication that can pass across the blood-brain barrier and has been effective in treating other tumor sites, it was an intriguing chemical to investigate for the treatment of metastatic breast cancer. Furthermore, as a cytotoxic alkylating agent it is chemically different compared to other drugs used to treat breast cancer. Due to these factors, the NCIC - Clinical Trials Group looked into TMZ's effectiveness in treating women whose breast cancer had spread and had previously received chemotherapy. To increase the likelihood of a response, a treatment plan of 150 mg/m2 on days 1-4 every two weeks (normal doses 86 every two weeks instead of every four weeks) was selected. Other phase II studies have investigated the activity of TMZ in patients with brain metastases, including those secondary to breast cancer 87-89. It has been demonstrated that TMZ in conjunction with cisplatin (CDPP), which decreases the DNA repair enzyme MGMT similarly to temozolomide, causes partial responses (PR) in breast cancer patients' extracranial and brain regions 87. In a phase II trial run by the Hellenic Cooperative Oncology Group, six out of fifteen women who were included achieved partial remission (PR) by using 150-200 mg/m2 on days 1-5 every 28 days with 75 mg/m2 of CDDP on day 1, including four patients who had progressed after receiving whole brain radiation therapy in the past. The same group's earlier phase II research 88, which assessed TMZ alone, was unable to show any improvements in patients with breast cancer. In a third research, 10 patients with breast cancer exhibited no response, whereas 4 individuals had stable brain disease for 8 weeks 89. The first investigation of single-agent TMZ in patients with breast cancer is this phase II trial. In order to ascertain the effectiveness and toxicity of TMZ in patients with metastatic breast cancer, Trudeau et al. carried out a phase 2 research in which a cohort of nineteen patients was administered a dosage dense regimen of 150 mg/m2 on days 1-7 and 15-21 in a 28-day cycle. These people with severely pretreated metastatic breast cancer, including brain metastases, did not show any response to TMZ 90. Hoffman et al. described the cases of two women with diffuse CNS metastases from breast cancer. Following irradiation of the symptomatic areas, TMZ 100 mg/m2 day 1-5/7 was administered in combination with intrathecal liposomal Ara-C every 2-4 weeks. Both patients' neurological symptoms and cerebrospinal fluid (CSF) cytology improved and stabilized over several months. After diagnosis, the patients lived for 10 and 17 months respectively, showing no symptoms of brain damage 91. The results of a phase I clinical trial in a cohort of women with metastatic HER2+ breast cancer to the brain following treatment with SRS or WBRT were published by Jenkins et al. in a relatively recent paper. Subsequently, the patients were administered a low-dose metronomic temozolomide together with an appropriate HER2-targeted systemic drug, T-DM1, to prevent brain metastases. Toxicities were mostly of low grade. Out of twelve patients, only two experienced new parenchymal brain metastases after an average follow-up of 9.6 months. The administration of temozolomide for the secondary prevention of brain metastases is supported for the first time by this trial 92.

### 6.4. Lung Cancer

Since temozolomide may pass across the blood-brain barrier in both animal and human models, it has demonstrated efficacy against brain metastases from a range of solid cancers, including NSCLC 88,89, 93. Moreover, TMZ has demonstrated some efficacy in treating NSCLC as a second-line treatment 94. Brain metastases are relatively common in NSCLC patients—nearly 20% at diagnosis and 40% at autopsy 95, 96. Since TMZ may be able to treat or prevent brain metastases, it may be a great option for these individuals. In a group of 31 NSCLC patients who had previously received treatment, Kouroussis et al. investigated the effectiveness of TMZ. Three patients (10%) had stable illness, and two patients (6.5%; 95% CI: -2.2 to 15.1%) had a partial response. The 1-year survival rate was 22.5%, the median survival time was 3.3 months, and the median time to progression was 2.4 months 97. TMZ did not exhibit any effect in NSCLC patients with or without brain metastases in an EORTC phase II investigation 98. Research on TMZ in patients with small cell lung cancer (SCLC) has a solid history. In SCLC, alkylating drugs are effective when used alone 99. Brain metastases are prevalent in this condition, and TMZ passes the blood-brain barrier 100. MGMT is abnormally methylated in SCLC 64, 101. Lastly, SCLC has reported anecdotal reactions to TMZ 102. In order to find out how effective TMZ was for patients with relapsed sensitive or refractory small cell lung cancer, Pietanza et al. carried out a phase II research. After one or two previous chemotherapy regimens, patients with disease progression were given TMZ at a dose of 75 mg/m2/d for 21 days within a 28-day cycle. In susceptible individuals, there was one CR and ten PRs [ORR, 23%; 95% confidence interval (CI), 12%-37%]. In the refractory cohort, two PRs were seen (ORR, 13%; 95% CI, 2%-38%). The ORR for second and third-line treatments, respectively, was 19% (95% CI, 7%-36%) and 22% (95% CI, 9%-40%). A CR or PR was present in 38% of patients with target brain lesions (95% CI: 14%-68%). In comparison to patients with unmethylated MGMT, a higher proportion of methylated MGMT cases (38% vs. 7%; P= 0.08) exhibited a reaction 103. Research has shown that when TMZ and WBRT were used together to treat patients with brain metastases from non-small cell lung cancer, the combination showed a greater response rate and a longer progression-free survival time 104. WBRT+TMZ can raise the ORR for brain metastases of NSCLC, according to a recent meta-analysis by Han et al. 105. However, there is an increased risk of treatment-associated grade III/IV hematological toxicity and gastrointestinal damage when compared to WBRT alone.

### 6.5. Prostate Cancer

Disappointing findings were found in a phase II research on TMZ and prostate cancer 106. The existence of aneuploid cell fractions, which provide a wide range of cells from extremely sensitive to medication resistant, may be one of the causes of this 107. Higher local TMZ concentrations were realized as a result of efforts to enhance this unsatisfactory state; these concentrations are adequate to kill cells regardless of inherent cellular sensitivity and cell DNA index. In order to restructure the TMZ for intervention, Braun et al. ligated it to a peptide-based carrier system known as TMZ-BioShuttle. The carrier is modular in nature, consisting of a transmembrane transporter (CPP) coupled to a cleavably-bound nuclear localization sequence (NLS) that was associated with TMZ. Following enzymatic cleavage within the cytoplasm and separation from the CPP, the TMZ-BioShuttle transmembrane passage and intracytoplasmic delivery of the TMZ into the cell nucleus are made possible by the NLS sequence. The hormone-refractory prostate cancer serves as an example of how this TMZ-BioShuttle may enhance treatment alternatives 108,109. Hussain et al. recently evaluated the safety and effectiveness of TMZ and veliparib (ABT-888), low dose oral PARP inhibitors, in patients with metastatic castration-resistant prostate cancer (mCRPC) who had received prior docetaxel treatment. Thirteen patients had stable PSA, ten had PSA advancement, and two had a verified PSA response (8.0 %; 95% CI: 1.0-26.0) 110.

### 6.6. Sarcomas

TMZ possesses anti-sarcoma properties similar to dacarbazine 111-113. Therefore, it might be helpful in treating metastases as well as primary control of sarcoma radiosensitization. In recurrent Ewing's sarcoma and desmoplastic small round cell tumors (DSRCT), Anderson et al. confirm a strong response rate that may even be higher than that documented in the literature 114-116. Compared to conventional regimens involving ifosfamide or cyclophosphamide, the combination of TMZ with irinotecan is less immunosuppressive 117. Given that it has been demonstrated that lymphocyte recovery—defined as an absolute lymphocyte count of more than 500 on day 15 following the first round of chemotherapy—is linked to a noticeably greater survival rate in Ewing's sarcoma, this may be particularly noteworthy in this case 118. Additionally, dacarbazine, commonly known as TMZ, has been used with other medications, such as doxorubicin liposomes 120 and gemcitabine 119. Temozolomide showed an objective response rate (ORR) of 18% when administered to patients with previously treated unresectable or metastatic leiomyosarcoma; 27% of patients experienced disease stabilization 121. Another phase II trial demonstrating an overall response rate of 15.5% involved 45 patients with soft-tissue sarcoma. Out of 11 patients with gynecologic leiomyosarcoma, 5 showed these responses 122. Noh et al. used mouse xenograft models and uterine sarcoma cell lines to assess the anticancer effects of cabozatinib, temozolomide, and their combination. They discovered that in uterine sarcoma cell lines and xenograft mice models, including PDX, cabozatinib and temozolomide together provide synergistic anticancer effects. These findings call for additional research in a phase 1 clinical trial.

### 6.7. Pediatric Tumors

Recent years have seen the completion of several TMZ trials on pediatric cancers. The Children's Cancer Group (CCG) carried out a phase I clinical trial with TMZ in children and young adults with recurrent solid tumors 124. The study's maximum tolerated dose (MTD) was 180 mg/m2/day for five days for patients who had previously received radiation therapy and 215 mg/m2/day for participants who had not received prior craniospinal irradiation (CSI). There was little evidence of major negative effects. Subsequent phase II research revealed TMZ activity in medulloblastoma and high-grade gliomas 125, among other forms of brain tumors. Sio et al. looked into the use of as a single agent in juvenile solid tumors that had relapsed or were resistant. For five days, the medication was given to patients who had previously received autologous bone marrow transplantation (ABMT) or craniospinal irradiation (CSI) at a dose of 215 mg/m2/day or 180 mg/m2/day, respectively. In our series, the objective response-rate (CR, PR, or MR) was 13.4% (1.9% CR, 3.8% PR, and 7.7% MR); 38.4% of patients experienced SD, and 48% had PD 126. 39 patients (median age B13 years; 14 pretreated with high-dose chemotherapy, craniospinal irradiation, or having bone marrow involvement) with refractory or recurrent solid tumors were evaluated by Geoerger et al. The patients received cisplatin treatment, followed by oral TMZ for five days every four weeks at dose levels of 80 mgm_2/150 mgm_2 day_1, 80/200, and 100/200, respectively. A total of 38 patients were eligible for toxicity evaluation (median 2, range 1-3). Two neuroblastomas, one brain stem glioma, and two malignant gliomas all showed partial responses. After five days of TMZ treatment, the median MGMT activity in PBMCs dropped; low MGMT activity was associated with a higher degree of thrombocytopenia. Combinations of cisplatin and temozolomide are well tolerated and do not cause any more harm than single-agent therapies 127. In 46 children with resistant solid tumors, Jakachi et al. performed a phase I and pharmacokinetic investigation of the epidermal growth factor receptor (EGFR) inhibitor erlotinib as a single drug and in combination with TMZ. Nineteen months were spent in stable condition for one patient with neurocytoma, twenty-three and twenty-four months were spent on study for two patients with neuroblastoma, and one patient with myoepithelioma saw a mixed response 128. In addition to being well tolerated, TMZ and irinotecan have been shown to be effective against a number of pediatric solid tumors, such as neuroblastoma 131 and Ewing sarcoma 129, 130. Wagner et al. examined the effectiveness of bevacizumab in combination with vincristine, oral irinotecan, and TMZ (VOIT Regimen) in pediatric patients with recurrent solid tumors or brain tumors. Tolerability was increased by reducing TMZ from 150 to 100 mg/m2/day; treatment with this reduced TMZ dose was practical and easy to administer as outpatient therapy. Even though Ewing sarcoma showed responses, it was uncertain whether adding bevacizumab would be beneficial 132. Temozolomide has been reported to be a successful treatment in a number of patients with metastatic PPGL (phaeochromocytoma/paraganglioma), according to two small studies 133,134 and several case reports 135. It has been demonstrated that patients with germline SDHB mutations responded more favorably to temozolomide 133,134.

## 7. Increasing the efficacy and overcoming the resistance of temozolomide in tumors

In order to increase the effectiveness of temozolomide, numerous tactics are being used that attempt to attack MGMT in various ways. Exosome-mediated circWDR62, miR-214-5p, and lncRNA UCA1/miR-182-5p have been shown to enhance resistance mechanisms to temozolomide 135,137. Patients with advanced hepatocellular carcinoma have been treated with temozolomide plus a MAPK/ERK inhibitor (U0126) because this combination increases the susceptibility of HCC cells to TMZ and down-regulates MGMT expression by blocking the MAPK/ERK signaling pathway 138. By ubiquitinating and degrading MGMT, TRIM72 improved the sensitivity of dacarbazine treatment 139, hence reinstating the resistance mechanism of dacarbazine treatment in uveal melanoma. Ultimately, it has been demonstrated that NCT503, Tubeimoside-I, GNA13, Pyrviniumpamoate, DEC1, METTL3, and MMR improve GBM sensitivity to TMZ treatment by controlling MGMT 140-146.

## 8. Parp-inhibitors and temozolomide combination

Preclinical data suggests that in MGMT-silenced tumors, this could improve tumor cell death 147-149. The multifaceted enhanced TMZ sensitivity of tumors with a PARPi takes use of PARP inhibitor activity in delaying the start of HR-mediated recovery 148. Additionally, the combination of temozolomide with PARP inhibitor sensitivity depends on "PARP trapping" on DNA, suggesting that olaparib is a molecule that can work in concert with temozolomide 148-151. Cechini et al. discovered that the combination of temozolomide and Olaparib was well tolerated by patients suffering from colorectal cancer, and that it did demonstrate anticancer effectiveness in a subgroup of patients whose tumors showed MGMT promoter hypermethylation, reduced MGMT protein expression, and enhanced CD8+ effector TILs. 152. O6-methylguanine (O6MeG), one of the several methyl adducts produced when exposed to temozolomide, makes up a small percentage of these adducts but is the main cytotoxic lesion that seriously hinders DNA replication because thymine is inserted in opposition to methylguanine 153,154. DNA mismatch repair (MMR), BER, the enzyme alkylpurine-DNA-N-glycosylase (APNG), or O6-methylguanine DNA methyltransferase (MGMT) can all be used to treat the O6MeG lesion. Temozolomide sensitivity is dependent on the expression of MGMT, APNG, and BER proteins in addition to MMR status 154. In a recent study, Drxheimer et al. investigated the potentiation of DNA-damaging drugs by pharmacologic modulation of DNA repair pathways using Multicellular Spheroids, an in vitro model of human solid tumors composed of malignant cells, endothelial cells, and mesenchymal stem cells. They discovered that when temozolomide and the PARP inhibitors olaparib and talazoparib were combined, there were clear synergistic effects 155. This is consistent with earlier preclinical research that showed temozolomide and PARP inhibitor worked synergistically in ten glioblastoma multiforme cancer stem cell lines 156. The more common N7MeG (N7-methylguanine) and N3MeA (N3-methyladenine) adducts are repaired by the BER pathway in a process that needs PARP activity, whereas MGMT reverses O6MeG lesions caused by cytotoxic temozolomide 157. Therefore, unrepaired and potentially fatal temozolomide-induced N7MeG and N3MeA lesions result from the suppression of PARP-mediated BER, which increases temozolomide cytotoxicity. Additionally, it has been shown that PARP's PARylation of MGMT is essential for the repair of O6MeG adducts, hence strengthening PARP's involvement in the BER and MGMT-mediated DNA repair of temozolomide-induced DNA damage 158. The temozolomide/PARP inhibitor combination has been and is still being evaluated in clinical trials for glioblastoma, SCLC, renal cancer, Ewing sarcoma, rhadomyosarcoma, and advanced stage rare cancers (NCT04434482, NCT04603365, NCT01858168), based on the encouraging results in preclinical cancer models 159-161.

## 9. Combining temozolomide with immunotherapy; is it an enhancer?

In addition to the direct effects on tumor cells discussed above, TMZ has also been demonstrated to have immunoregulatory qualities. Like all other chemotherapy drugs, TMZ also has a variety of effects on the immune system, but maybe the most significant one is that it modifies the characteristics of immune cells, particularly the ratio of Treg cells to T-cells and the proliferation of T-cells 162. Since TMZ has been the most commonly used treatment for GBM patients, the most research has been done on the immunological changes it makes to the tumor microenvironment (ME) and in a systematic manner. The most frequently reported systemic immunological response is lymphopenia. Numerous investigations have revealed a considerable decline in lymphocytes, particularly B-cells and CD4+ T-cells, and to a lesser extent, CD8+ T-cells 162, 163. From a therapeutic standpoint, these systemic effects of TMZ have been examined in a few clinical trials where cellular immunotherapy drugs were also provided with TMZ. One of them used TMZ as an adjuvant in conjunction with a peptide vaccination targeting the EGFRvIII mutant version of the epidermal growth factor receptor. This trial's underlying mechanism relates to the immune-stimulatory effects it likely possesses, aside from its cytotoxic qualities, as it has been demonstrated to increase the tumor cells' susceptibility to T-cell death and phagocytosis. This happens as a result of TMZ's upregulation of calreticulin (CRT) surface expression and its forced release of high-mobility group 1 protein (HMGB1). Additionally, both molecules have danger-associated molecular pattern molecules (DAMP) and function as DC and macrophage stimulants. These qualities have the potential to increase immune responses against tumors, hence reducing the need for additional adjuvants. In vivo research has demonstrated favorable results in terms of long-term survival 163-165. According to the previously described research, there was a notable increase in the number of regulatory T-cells in response to the tumor cells, indicating that TMZ was more than just a chemotherapeutic drug 164. TMZ has an impact on GBM immunological ME as well. Since the GBM ME is known to be extremely immunosuppressive due to its excretion of IL-11, which improves the tumor cells' ability to evade the immune system through the STAT3-MYC pathway 165. Tregs, myeloid-derived suppressor cells (MDSCs), and macrophages are the immunosuppressive components that make up the ME. As was previously mentioned, TMZ systematically reduces the amount of Tregs. However, TMZ alone is unable to counteract the immunosuppressive characteristics of ME and change its immunological characteristics. Overall, TMZ continues to play a significant role in the treatment of GBM, either as an immune system modulator or as a cytotoxic agent. The combination of TMZ with other immunotherapy agents, such as immune checkpoint inhibitors, is an area that warrants further investigation and further study 162,165.

**Figure 3:**
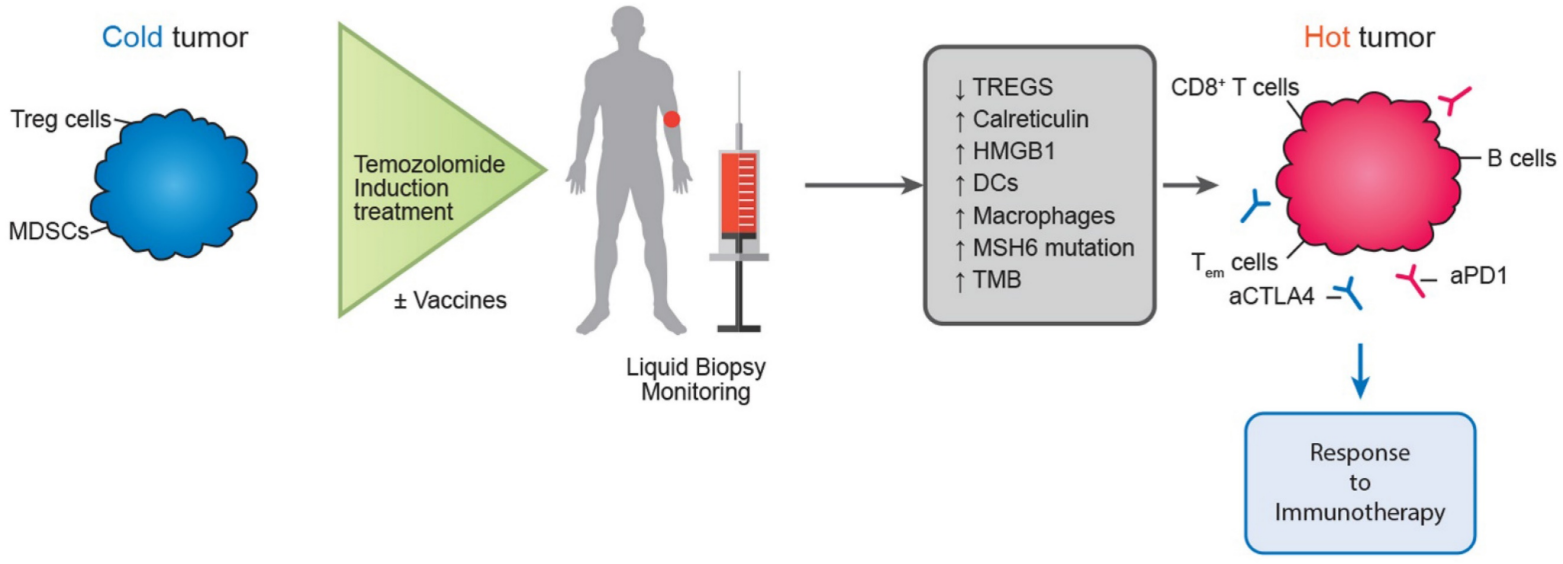
Potential future role of temozolomide in turning immune “cold” tumors to “hot” ones.

In a similar direction, Morano et al. carried out the phase II MAYA trial in patients with metastatic colorectal cancer, attempting to take advantage of the immunomodulatory benefits of temozolomide. The MAYA trial concluded by presenting data regarding the function of temozolomide as an immune-sensitizing drug for immune-cold mCRCs and MSS that are chosen based on MGMT silencing and disease control during temozolomide priming. In patients with microsatellite-stable (MSS) and O6-methylguanine-DNA methyltransferase (MGMT)-silenced metastatic colorectal cancer (mCRC), the researchers administered two cycles of temozolomide first as an immunosensitizer, and then a combination of low-dose ipilimumab and nivolumab. There was evidence of clinical benefit in this patient group, thereby proving the theory that temozolomide priming followed by a combination of low-dose ipilimumab and nivolumab may produce long-lasting clinical benefit in MSS and MGMT silenced mCRC 166. Researchers included colorectal cancer patients with O6-methylguanine-DNA-methyltransferase (MGMT)-deficient tumors that were also MMR-proficient and RAS-mutant in the Arethusa trial, a proof of concept trial that involved priming therapy with TMZ. Following TMZ therapy, a unique mutational signature and elevated TMB were found by analysis of tissue samples and circulating tumor DNA (ctDNA). MMR genes showed several changes in the nucleotide context preferred by the TMZ signature, and in 94% (16/17) of the patients, the p.T1219I MSH6 variant was found in the ctDNA and tissue. After receiving pembrolizumab treatment, a subgroup of patients whose tumors had the TMZ mutational profile, elevated TMB, and the MSH6 mutation had stable disease 167. The proof of concept for temozolomide's possible function in converting immunological "cold" tumors to "hot" ones, where immunotherapy may then be therapeutically advantageous, was made feasible by these two trials (Figure 3).

## 10. Combining Temozolomide with Radiotherapy

The current standard treatment for patients with newly diagnosed GBM involves targeted radiation therapy together with chemotherapy, followed by additional cycles of chemotherapy as per the Stupp regimen. This approach was established by the EORTC-NCIC phase 3 trial around twenty years ago 71,168. RT and TMZ cause an accumulation of DNA damage in the form of single-stranded breaks (SSBs) or double-stranded breaks (DSBs) leading to tumor cell death. Hegi et al. found that the impact on survival was particularly significant in glioblastoma patients with MGMT promoter methylation when combining radiation with temozolomide. Patients with glioblastoma and unmethylated MGMT promoters did not have improved survival when temozolomide was added to radiation treatment. The 2-year survival rates for the four patient groups, categorized by unmethylated and methylated MGMT promoters and treated with either radiation alone or radiotherapy combined with temozolomide, are 2%, 14%, 23%, and 46%, respectively 21. The effect of prolonged adjuvant TMZ treatment (more than 6 cycles) on survival results has been a topic of debate with no agreement on the best duration of adjuvant TMZ therapy 169, 170. MGMT gene promoter methylation is used as a predictive marker for response to alkylating TMZ chemotherapy. Some oncologists are extending adjuvant TMZ treatment beyond the standard 6 cycles, up to 12 or even 24 cycles, based on personal or institutional preferences, despite the lack of solid scientific evidence regarding its efficacy and safety. In order to further enhance the activity of the combination of Temozolomide and radiotherapy, an interesting phase 1 trial is underway, that intracranially administers γδ T cells modified to be temozolomide resistant so as to be active, concurrent with temozolomide and radiotherapy, a strategy called “Drug Resistance Immunotherapy”

## 11. Conclusions

Temozolomide has exhibited activity to various solid tumors. Due to its advantageous pharmacodynamic profile and to new combinations that overcome the phenomenon of chemoresistance, broaden its use as an active molecule in advanced cancers where effective treatments are on demand.

## Figures and Tables

**Table 1:** Trials with Temozolomide combinations in solid tumors.

INVESTIGATORS	CLINICAL TRIAL ID	ENROLLING PATIENTS (E) OR COMPLETED (C)	COMBINED MOLECULE	PHASE	TYPE OF STUDY	ENDPOINT	CHILDREN (UNTIL 18Y) (C) OR ADULT (A)	NEOPLASM	TEMODAL DOSE AND SCHEDULE
Bhardwaj Dessai, MD,	NCT01193140	C	VELIPARIB	2	Non-Randomized, Open Labeled	SAFETY	A	SOLID TUMORS	Dose orally once daily for 5 days, consecutively, every cycle
Jeffrey A. Sosman, MD	NCT00512798	C	BORTEZOMIB	1,2	Non-Randomized, Open Label, Single Group Assignment	DOSE AND EFFICACY	A	SOLID TUMORS, MELANOMA	PHASE 1: 50, 75mg/m2 PHASE 2: 75 mg/m2 po daily, during weeks 2-8 (42 days) of every 9-week course.
Matthew Taylor, MD, Antonio Omuro, MD	NCT01107522	E	carboxyamidotriazole orotate (CTO)	1	Interventional	SAFETY, TOLERABILITY, AND THE MAXIMUM TOLERATED DOSE/RECOMMENDED PHASE II DOSE	A	Glioblastoma, Recurrent Malignant Gliomas,Solid Tumors	ARM B: orally at fixed dose of 150 mg/m2 daily for Days 1-5 /28dARM C: po 75 mg/m2/d during RT, then at 150mg/m2 for 1-5 d of Cycle 1, and then up to 200 mg/m2 1-5d of subsequent cycles
Jennifer Eads, MD	NCT00892385	E	METHOXYAMINE	1	Interventional, Open Label, Single Group Assignment	SAFETY/EFFICACY	A	Advanced solid tumours	Tem per os/d 1-5d. every 28 days
Rochelle Bagatell	NCT01141244	C	TEMSIROLIMUS, IRINOTECAN	1	Interventional, Open Label, Single Group Assignment	SAFETY	C	RECURRENT OR REFRACTORY SOLID TUMOURS	temsirolimus IV over 30 minutes on days 1 and 8 or on days 1, 8, and 15 and temozolomide PO and irinotecan hydrochloride PO on days 1-5
Shivaani Kummar, M.D.	NCT01851369	E	TRC102	1	Interventional, Open Label, Single Group Assignment, Non-randomized	SAFETY, EFFICACY	A	ADVANCED SOLID TUMORS OR LYMPHOMAS	TEM po 1-5days
Jana Portnow, MD	NCT00544284	C	BORTEZOMIB	1	Interventional, Open Label, Single Group Assignment, Non-randomized	SAFETY	A	REFRACTORY SOLID TUMORS, BRAIN CNS TUMORS, LYMPHOMA	TEM po 1-5days
Zev Wainberg	NCT02049593	E	(PARP) inhibitor BMN-673, OR IRINOTECAN ALONE	1	Interventional, Open Label, Non-randomized	SAFETY, DOSE ESCALATION	A	ADVANCED SOLID TUMORS	TEM po 1-5days
Lars M. Wagner, MD, John P. Perentesis, MD	NCT00138216	C	VINCRISTINE, IRINOTECAN	1	Interventional, Open Label, Single Group Assignment	SAFETY, DOSE ESCALATION	C & A TILL 21Y	BRAIN AND CNS TUMORS, SOLID TUMORS	TEM po 1-5days
Eisai Medical Services	NCT01127178	C	(PARP) Inhibitor E7016	1	Interventional, Open Label, Single Group Assignment	SAFETY, DOSE ESCALATION	A	ADVANCED SOLID TUMORS AND GLOMAS	TEM po 1-5days
Pamela Z New, M.D.	NCT01736800	E	TOPOTECAN	2	Interventional, Open Label, Single Group Assignment	SAFETY/EFFICACY	A	SOLID TUMORS WITH CNS METASTASES	TEM po 1-5days
Lionel D. Lewis, MD	NCT00014261	E	PEG-interferon alfa-2B	1	Interventional	EFFICACY, DOSE ESCALATION	A	Refractory And/Or Advanced Solid tumors	TEM on days 1-7 and 15-21
Elizabeth Fox, MD, Holly Meany, MD	NCT00303940	C	TALABOSTAT	1	Interventional	SAFETY, DOSE ESCALATION	C	RELAPSED OR REFRACTORY BRAIN TUMORS, SOLID TUMORS	TEM po 1-5days
Katherine Warren, MD	NCT00020150	C	O6-benzylguanine	1	Interventional	EFFICACY	C & A (UP TO 21Y)	SOLID TUMORS	TEM po 1-5days
Damon Reed, M.D., Jonathan Gill, M.D.	NCT01528046	E	Metformin, Irinotecan, Vincristine	1	Interventional, Open Label, Single Group Assignment	SAFETY/EFFICACY	C	RECCURENT REFRACTORY SOLID TUMORS	TEM:100 mg/m^2/day PO Days 1-5
Rajkumar Venkatramani, MD	NCT00993044	C	Irinotecan, Vincristine, Bevacizumab	1	Interventional, Open Label, Single Group Assignment	SAFETY	C	REFRACTORY SOLID TUMORS	TEM:100 mg/m^2/day PO Days 1-5
Eli Lilly and Company	NCT01284335	C	LY573636-sodium	1	Interventional, Open Label,Non-Randomized	SAFETY, EFFICACY	A	ADVANCED SOLID TUMORS	200 mg/m2 administered orally on days 1-5 of a 28 day cycle
Brian Turpin, D.O.	NCT00786669	C	Bevacizumab,vincristine, irinotecan	1,2	Interventional, Open Label, Single Group Assignment	SAFETY, EFFICACY	C & A (UP TO 30Y)	RELAPSED OR REFRASCTORY SOLID TUMORS	00 mg/m2/day po on Days 1-5 every 3 weeks for up to 6 cycles
Sponsor's Medical Expert, MD	NCT00920595	C	CEP-9722	1	Interventional, Open Label, Single Group Assignment	SAFETY, EFFICACY	A	ADVANCED SOLID TUMORS	TEMO 150 mg/m2/day on Days 1-5
Regina Jakacki	NCT00077454	C	ERLOTINIB	1	Interventional, Open Label, Single Group Assignment	SAFETY	A	RECCURENT/REFRACTORY SOLID TUMORS	TEM po 1-5days
Judith M. Ford, MD, PhD	NCT00012116	C	NO	2	Interventional, Open Label, Single Group Assignment	EFFICACY	A	ADVANCED SOLID TUMORS WITH BRAIN METS	once a day for 6 weeks followed by 4 weeks of rest/ Daily dose: 75mg/m2.
Eric Schafer	NCT02116777	C	PARP INHIBITOR BMN-673	1,2	Interventional, Open Label, Single Group Assignment	SAFETY, EFFICACY, DOSE ESCALATION	C& A (UP TO 30 Y)	REFRACTORY OR RECURRENT MALIGNANCIES	TEMO PO QD on days 2-6/28days
Stanton L. Gerson, MD	NCT00003567	C	Mutant MGMT Gene Transfer Into Human Hematopoietic Progenitors, O6-Benzylguanine, carmustine	1	Interventional, Open Label, Single Group Assignment	SAFETY, EFFICACY	A	ADVANCED SOLID TUMORS- NON-HODGKIN LYMPHOMAS	Four weeks after the completion of BG and carmustine, patients receive TEMO IV over 1 hour every 4 weeks for up to 5 courses,
Cynthia E. Herzog, MD	NCT00492141	C	liposomal 9-Nitro-20-(S)-Camptothecin (L9-NC) by aerosol	1,2	Interventional, Open Label, Single Group Assignment/ Non-Randomized	SAFETY, EFFICACY	A	EWING'S SARCOMA AND SOLID TUMORS INVOLVING THE LUNG	100 mg/m^2 oral/day for Cycle 2 Days 1-5.
Merck Sharp & Dohme Corp.	NCT00960063	C	SCH 717454, Irinotecan	1/1B	Non-randomized, open-label, dose-escalation study	SAFETY	C&A (UP TO 21Y)	SOLID TUMORS	TEMO 100 mg/m2/day on Days 1-5
Henry S. Friedman, MD	NCT00005952	C	given with peripheral stem cell transplantation	1,2	Interventional	SAFETY, DOSE ESCALATION	C	MALIGNANT GLIOMAS, RECURRENT CNS TUMORS, SOLID TUMORS	oral temozolomide daily for 5 consecutive days
Ruth Plummer, Prof	NCT01618136	E	Polymerase (PARP) Inhibitor E7449	1,2	Non-randomized, open-label, dose-escalation study	SAFETY, DOSE ESCALATION, EFFICACY	A	ADVANCED SOLID TUMORS, OR B-CELL MALIGNANCIES	150 mg/m2/d TMZ administered orally, once daily for 5 days /28d
Volker W. Stieber, MD	NCT00049361	C	WBRT and Thalidomide	2	Interventional, Open Label	EFFICACY	A	SOLID TUMORS WITH BRAIN METS	Beginning on the day before the first radiation treatment, patients receive oral thalidomide once daily and oral temozolomide once daily for 21 days.
Thomas H. Davis, MD	NCT00005812	C	NO	2	Intereventional, Open Label, Single Group Assignment	SAFETY/EFFICACY	A	LEPTOMENINGEAL METASTASES FROM SOLID TUMORS OR LYMPHOMA	Oral temozolomide 75 mg/m2/day for 6 weeks, followed by 4 week break
William H Meyer, MD	NCT00222443	C	IRINOTECAN, VINCRISTINE, VANTIN	1	Intereventional, Open Label, Parallel Assignment, Non-Randomized	SAFETY/EFFICACY	C&A (up TO 21Y)	RECURRENT SOLID TUMORS OR LYMPHOMAS	Temozolomide is given by mouth one hour prior to each daily irinotecan dose days 1-5 of each cycle. 100 mg/m2/day.
Robert Bukowski	NCT00401180	C	DOCETAXEL	1	Intereventional, Open Label, Single Group Assignment	SAFETY, DOSE ESCALATION	A	METASTATIC SOLID TUMORS	orally daily for 3 weeks (escalating doses of 75 to 100 mg/m(2))
Bhardwaj Desai, MD	NCT00526617	C	ABT-888	1	Intereventional, Open Label, Single Group Assignment	SAFETY	A	SOLID TUMORS, etastatic melanoma (MM), BRCA deficient breast, ovarian, primary peritoneal, or fallopian tube cancer, and hepatocellular carcinoma (HCC).	-
Sanofi	NCT00422682	C	BSI-201	1B	Intereventional, Open Label, Parallel Assignment	SAFETY/EFFICACY	A	ADVANCED SOLID TUMORS	-
Merck Sharp & Dohme Corp.	NCT01294735	C	MK-4827	1	Intereventional, Open Label, Parallel Assignment	SAFETY/EFFICACY	A	ADVANCED CANCER	-
Shivaani Kummar, M.D	NCT01827384	E	OR EVEROLIMUS OR CARBOPLATIN OR TRAMETINIB DMSO OR ABT-888 OR MK-1775	2	Intereventional, Open Label, Parallel Assignment, Non-Randomized	EFFICACY	A	ADVANCED SOLID TUMORS	-
Fa-Chyi Lee, MD	NCT00249964	C	PACLITAXEL, CARBOPLATIN	1	Intereventional, Open Label, Single Group Assignment, Non-Randomized	EFFICACY, DOSE ESCALATION	A	SOLID TUMORS	starting dose of temozolomide at 75 mg/m2/day from day 2 to day 6, a total of 5 days/21days
Sajeel Chowdhary, MD, Jade Homsi, MD	NCT00437957	C	VALPROIC ACID, RT	1	Intereventional, Open Label, Single Group Assignment	SAFETY/EFFICACY	A	SOLID TUMORS WITH BRAIN METS	75 Mg/m2/day for all Cohorts
Michael J Pishvaian, MD, PhD	NCT01051596	C	ABT-888	1	Open Label, Single Group Assignment, Non-Randomized	EFFICACY	A	ADVANCED COLORECTAL CANCER	150 mg/m2 once a day on Days 1-5 of each 28-day cycle
Pamela Kunz	NCT01824875	E	CAPECITABINE	2	Interventional, Randomized, Parallel Assignement, Open Label	EFFICACY	A	ADVANCED PANCREATIC NEUROENDOCRINE TUMORS	ARM A:TEMO PO QD on days 1-5./28d, ARM B: TEMO PO QD on days 10-14/28d
Suman Malempati	NCT01055314	C	ETOPOSIDE, VINCRISTINE, IRINOTECAN, IFOSFAMIDE, DOXORUBICIN, CYCLOPHOSHAMIDE, DACTINOMYCIN, RT	1	Interventional, Randomized, Open Label, Parallel Assignment	SAFETY/EFFICACY	C& A (up to 49 y)	METASTATIC RHABDOMYOSARCOMA	TEMO PO on days 1-5 of weeks 1, 4, 20, 23, 47, and 50.
Morris D. Groves, MD	NCT00515788	C	intrathecal liposomal cytarabine (DepoCyt)	1	Intereventional, Open Label, Single Group Assignment	SAFETY/EFFICACY	A	SOLID TUMORS, LYMPHOMA WITH NEOPLASTIC MENINGITIS	100 mg/m2 po daily for 7 days every 14 days.
Philipp Hoffmanns, MD, PhD	NCT02231762	E	Lanreotide Autogel 120 mg	1	Intereventional, Open Label, Single Group Assignment	EFFICACY	A	Progressive Gastro-entero-pancreatic Neuroendocrine Tumours (GEP-NET) G1/G2	TEMO PO 150 mg/m2 per day for 5 days in the first month. 200 mg/m2 per day for 5 days in months 2, 3, 4, 5 and 6.
Carlos Gamboa-Vignolle, MD	NCT01015534	C	WBRT	2	Interventional, Randomized, Parallel Assignement, Open Label	EFFICACY	A	SOLID TUMORS WITH BRAIN METS	1h before each fraction of whole brain irradiation, 200 mg on Monday, Wednesday, Friday; 300 mg on Tuesday, and Thursday.
Merck Sharp & Dohme Corp	NCT00034697	C	RT	2	Double-blind Interventional, Randomized, Parallel Assignement, Open Label	SAFETY/EFFICACY	A	NSCLC WITH BRAIN METS	-
Hoffmann-La Roche	NCT00811993	C	R1507	1	Interventional, Randomized, Parallel Assignement, Open Label	SAFETY	A	ADVANCED MALGINANT NEOPLASMS	AS PRESCRIBED
Oana C Danciu, M.D	NCT03332355	C	PAC-1	1	Interventional Single group assignment	SAFETY, DOSE ESCALATION	A	ADVANCED SOLID TUMOR OR HEMATOLOGIC MALIGNANCY (LIMITED TO LYMPHOMA)	150 MG/M2 DOSE OF TEMOZOLOMIDE GIVEN FOR THE 5 DAYS STARTING AT DAY 8 OF CYCLE 1
Wen-Jen Hwu, MD, PhD,	NCT00005815	C	Thalidomide	1,2	Interventional	SAFETY, DOSE ESCALATION	A	METASTATIC MALIGNANT MELANOMA THAT IS CONSIDERED UNRESECTABLE STAGE III OR IV OCULAR, MUCOSAL, OR CUTANEOUS MELANOMA	ESCALATING DOSES OF TEMOZOLOMIDE UNTIL THE MAXIMUM TOLERATED DOSE (MTD) IS DETERMINED
Novartis Pharmaceuticals	NCT05429502	E	Ribociclib (LEE011) in Combination With Topotecan	1,2	Randomized parallel assignment	SAFETY/EFFICACY	C&A (up TO 21Y)	Neuroblastoma, Medulloblastoma, High-grade glioma, Malignant rhabdoid tumor, Rhabdomyosarcoma	TEMOZOLAMIDE ADMINISTERED AT THE STANDARD DOSE GIVEN TO NEUROBLASTOMA PATIENTS
María Angeles Vaz, M.D	NCT03466450	E	Glasdegib (SHH pathway inhibitor)	1,2	Phase Ib/II, multicentric, non-randomized, open label	SAFETY/EFFICACY	A	GBM	TMZ at 75 mg/m2 /d concurrently with RT for a maximum of 42 days. At 4 weeks after RT completion, patients will start taking TMZ at 150 mg/m2/d for the first 5 days of a 28-day cycle. If first cycle is well tolerated, patients will receive TMZ at 200 mg/m2/d for the first 5 days of every subsequent 28-day cycle for another 5 cycles.

## References

[1] Middleton MR, Grob JJ, Aaronson N, Fierlbeck G, Tilgen W, Seiter S, Gore M, Aamdal S, Cebon J, Coates A, Dreno B, Henz M, Schadendorf D, Kapp A, Weiss J, Fraass U, Statkevich P, Muller M, Thatcher N (2000). Randomized phase III study of temozolomide versus dacarbazine in the treatment of patients with advanced metastatic malignant melanoma. J Clin Oncol.

[2] D'Atri S, Tentori L, Lacal PM, Graziani G, Pagani E, Benincasa E, Zambruno G, Bonmassar E, Jiricny J Involvement of the mismatch repair system in temozolomide-induced apoptosis Mol Pharmacol. 1998; 54:334-341.

[3] Danson SJ, Middleton MR (2001). Temozolomide: a novel oral alkylating agent. Expert Rev Anticancer Ther.

[4] Newlands ES, Stevens MF, Wedge SR, Wheelhouse RT, Brock C Temozolomide (1997). a review of its discovery, chemical properties, pre-clinical development and clinical trials. Cancer Treat Rev.

[5] Stevens MF, Hickman JA, Langdon SP, Chubb D, Vickers L, Stone R, Baig G, Goddard C, Gibson NW, Slack JA, Newton C, Lunt E, Fizames C, Lavelle F (1987). Antitumor activity and pharmacokinetics in mice of 8-carbamoyl-3-methyl-imidazo[5, 1-d]-1, 2, 3, 5-tetrazin-4(3H)-one (CCRG 81045; M & B 39831), a novel drug with potential as an alternative to dacarbazine. Cancer Res.

[6] Bull VL, Tisdale MJ (1987) Antitumour imidazotetrazines-XVI (1987). Macromolecular alkylation by 3-substituted imidazotetrazinones. Biochem Pharmacol.

[7] Tisdale MJ (1987). Antitumor imidazotetrazines-XV. Role of guanine O6 alkylation in the mechanism of cytotoxicity of imidazotetrazinones. Biochem Pharmacol.

[8] Karran P, Offman J, Bignami M (2003). Human mismatch repair, drug-induced DNA damage, and secondary cancer. Biochimie.

[9] Ochs K, Kaina B (2000). Apoptosis induced by DNA damage O6-methylguanine is Bcl-2 and caspase-9/3 regulated and Fas/caspase-8 independent. Cancer Res.

[10] Kaina B, Ziouta A, Ochs K, Coquerelle T (1997). Chromosomal instability, reproductive cell death and apoptosis induced by O6-methylguanine in Mex-, Mex+ and methylation-tolerant mismatch repair compromised cells: facts and models. Mutat Res.

[11] Karran P, Bignami M (1994). DNA damage tolerance, mismatch repair and genome instability. Bioessays.

[12] Roos WP, Kaina B (2006). DNA damage-induced cell death by apoptosis. Trends Mol Med.

[13] Hirose Y, Berger MS, Pieper RO (2001). p53 effects both the duration of G2/M arrest and the fate of temozolomide-treated human glioblastoma cells. Cancer Res.

[14] Denny BJ, Wheelhouse RT, Stevens MF (1994). NMR and molecular modeling investigation of the mechanism of activation of the antitumor drug temozolomide and its interaction with DNA. Biochemistry.

[15] Hammond LA (1999). A Phase I and pharmacokinetic study of temozolomide on a daily _ 5 schedule in patients with advanced solid malignancies. J Clin Oncol.

[16] Bleehen NM (1995). Cancer Research Campaign Phase II trial of temozolomide in metastatic melanoma. J Clin Oncol.

[17] Yung WK (1999). Multicenter Phase II trial of temozolomide in patients with anaplastic astrocytoma or anaplastic oligoastrocytoma at first relapse. J Clin Oncol.

[18] Friedman HS (1998). DNA mismatch repair and O6-alkylguanine-DNA alkyltransferase analysis and response to Temodal in newly diagnosed malignant glioma. J Clin Oncol.

[19] Friedman HS (1995). Activity of temozolomide in the treatment of central nervous system tumour xenografts. Cancer Res.

[20] Esteller M, Garcia-Foncillas J, Andion E, Goodman SN, Hidalgo OF (2000). Inactivation of the DNA-repair gene MGMT and the clinical response of gliomas to alkylating agents. N Engl J Med.

[21] Hegi ME, Diserens AC, Gorlia T, Hamou MF, de Tribolet N (2005). MGMT gene silencing and benefit from temozolomide in glioblastoma. N Engl J Med.

[22] Palmisano WA, Divine KK, Saccomanno G, Gilliland FD, Baylin SB (2000). Predicting lung cancer by detecting aberrant promoter methylation in sputum. Cancer Res.

[23] Anda T, Shabani HK, Tsunoda K, Tokunaga Y, Kaminogo M (2003). Relationship between expression of O6-methylguanine-DNA ethyl transferase, glutathione-S-transferase pi in glioblastoma and the survival of the patients treated with nimustine hydrochloride: an immunohistochemical analysis. Neurol Res.

[24] Chinot OL, Barrie M, Fuentes S, Eudes N, Lancelot S (2007). Correlation between O6-methylguanine-DNA methyltransferase and survival in inoperable newly diagnosed glioblastoma patients treated with neoadjuvant temozolomide. J Clin Oncol.

[25] Pollack IF, Hamilton RL, Sobol RW, Burnham J, Yates AJ (2006). (2006) O6-methylguanine-DNA methyltransferase expression strongly correlates with outcome in childhood malignant gliomas: results from the CCG-945 Cohort. J Clin Oncol.

[26] Capper D, Mittelbronn M, Meyermann R, Schittenhelm J (2007) Pitfalls in the assessment of MGMT expression, in its correlation with survival in diffuse astrocytomas (2008). proposal of a feasible immunohistochemical approach. Acta Neuropathol (Berl).

[27] Grasbon-Frodl EM, Kreth FW, Ruiter M, Schnell O, Bise K (2007). Intratumoral homogeneity of MGMT promoter hypermethylation as demonstrated in serial stereotactic specimens from anaplastic astrocytomas and glioblastomas. Int J Cancer.

[28] Preusser M, Janzer RC, Felsberg J, Reifenberger G, Hamou MF (2008). Anti-O6-Methylguanine-Methyltransferase (MGMT) Immunohistochemistry in Glioblastoma Multiforme: Observer Variability and Lack of Association withPatient Survival Impede Its Use as Clinical Biomarker. Brain Pathol.

[29] Brell M, Tortosa A, Verger E, Gil JM, Vinolas N (2005). (2005) Prognostic significance of O6-methylguanine-DNA methyltransferase determined by promoter hypermethylation and immunohistochemical expression in anaplastic gliomas. Clin Cancer Res.

[30] Stupp R, Hegi ME Methylguanine methyltransferase testing in glioblastoma (2007). when and how?. J Clin Oncol.

[31] Bearzatto A, Szadkowski M, Macpherson P, Jiricny J, Karran P Epigenetic regulation of the MGMT, hMSH6 DNA repair genes in cells resistant to methylating agents Cancer Res. 2000; 60: 3262-3270.

[32] Grombacher T, Mitra S, Kaina B (1996). Induction of the alkyltransferase (MGMT) gene by DNA damaging agents and the glucocorticoid dexamethasone and comparison with the response of base excision repair genes. Carcinogenesis.

[33] Fritz G, Tano K, Mitra S, Kaina B (1991). Inducibility of the DNA repair gene encoding O6-methylguanine-DNA methyltransferase in mammalian cells by DNA-damaging treatments. Mol Cell Biol.

[34] Paz MF, Yaya-Tur R, Rojas-Marcos I, Reynes G, Pollan M (2004). CpG island hypermethylation of the DNA repair enzyme methyltransferase predicts response to temozolomide in primary gliomas. Clin Cancer Res.

[35] Weller M, van den Bent M, Preusser M, Le Rhun E, Tonn JC, Minniti G (2021). EANO guidelines on the diagnosis and treatment of diffuse gliomas of adulthood. Nature Reviews Clinical Oncology.

[36] Sasai K, Nodagashira M, Nishihara H, Aoyanagi E, Wang L, Katoh M (2008). Careful exclusion of non-neoplastic brain components is required for an appropriate evaluation of O6-methylguanine-DNA methyltransferase status in glioma: relationship between immunohistochemistry and methylation analysis. The American Journal of Surgical Pathology.

[37] Quillien V, Lavenu A, Ducray F, Joly MO, Chinot O, Fina F (2016). Validation of the high-performance of pyrosequencing for clinical MGMT testing on a cohort of glioblastoma patients from a prospective dedicated multicentric trial. Oncotarget.

[38] Xie H, Tubbs R, Yang B (2015). Detection of MGMT promoter methylation in glioblastoma using pyrosequencing. International Journal of Clinical and Experimental Pathology.

[39] Nguyen N, Redfield J, Ballo M, Michael M, Sorenson J, Dibaba D (2021). Identifying the optimal cutoff point for MGMT promoter methylation status in glioblastoma. CNS Oncology.

[40] Hosoya T, Takahashi M, Honda-Kitahara M, Miyakita Y, Ohno M, Yanagisawa S (2022). MGMT gene promoter methylation by pyrosequencing method correlates volumetric response and neurological status in IDH wild-type glioblastomas. Journal of Neuro-Oncology.

[41] Jahin M, Fenech-Salerno B, Moser N, Georgiou P, Flanagan J, Toumazou C (2021). Detection of MGMT methylation status using a Lab-on-Chip compatible isothermal amplification method. Annual International Conference of the IEEE Engineering in Medicine and Biology Society. IEEE Engineering in Medicine and Biology Society. Annual International Conference.

[42] von Rosenstiel C, Wiestler B, Haller B, Schmidt-Graf F, Gempt J, Bettstetter M (2020). Correlation of the quantitative level of MGMT promoter methylation and overall survival in primary diagnosed glioblastomas using the quantitative MethyQESD method. Journal of Clinical Pathology.

[43] Hegi ME, Genbrugge E, Gorlia T, Stupp R, Gilbert MR, Chinot OL (2019). MGMT Promoter Methylation Cutoff with Safety Margin for Selecting Glioblastoma Patients into Trials Omitting Temozolomide: A Pooled Analysis of Four Clinical Trials. Clinical Cancer Research.

[44] McGarry SD, Hurrell SL, Kaczmarowski AL, Cochran EJ, Connelly J, Rand SD (2016). Magnetic Resonance Imaging-Based Radiomic Profiles Predict Patient Prognosis in Newly Diagnosed Glioblastoma Before Therapy. Tomography.

[45] Salihoğlu YS, Uslu Erdemir R, Aydur Püren B, Özdemir S, Uyulan Ç, Ergüzel TT (2022). Diagnostic Performance of Machine Learning Models Based on 18F-FDG PET/CT Radiomic Features in the Classification of Solitary Pulmonary Nodules. Molecular Imaging and Radionuclide Therapy.

[46] Le NQK, Do DT, Chiu FY, Yapp EKY, Yeh HY, Chen CY (2020). XGBoost Improves Classification of MGMT Promoter Methylation Status in IDH1 Wildtype Glioblastoma. Journal of Personalized Medicine.

[47] Do DT, Yang MR, Lam LHT, Le NQK, Wu YW (2022). Improving MGMT methylation status prediction of glioblastoma through optimizing radiomics features using genetic algorithm-based machine learning approach. Scientific Reports.

[48] Lu J, Li X, Li H (2021). Perfusion parameters derived from MRI for preoperative prediction of IDH mutation and MGMT promoter methylation status in glioblastomas. Magnetic Resonance Imaging.

[49] Yogananda CGB, Shah BR, Nalawade SS, Murugesan GK, Yu FF, Pinho MC (2021). MRI-Based Deep-Learning Method for Determining Glioma MGMT Promoter Methylation Status. American Journal of Neuroradiology.

[50] Weaver KD, Grossman SA, Herman JG (2006). Methylated tumor specific DNA as a plasma biomarker in patients with glioma. Cancer Investigation.

[51] Fiano V, Trevisan M, Trevisan E, Senetta R, Castiglione A, Sacerdote C (2014). MGMT promoter methylation in plasma of glioma patients receiving temozolomide. Journal of Neuro-Oncology.

[52] Selzer E, Schlagbauer-Wadl H, Okamoto I, Pehamberger H, Pötter R, Jansen B (1998). Expression of Bcl-2 family members in human melanocytes, in melanoma metastases and in melanoma cell lines. Melanoma Res.

[53] Beier D, Schulz JB, Beier CP Chemoresistance of glioblastoma cancer stem cells-much more complex than expected Mol Cancer. 2011;10:128.

[54] Yip S, Miao J, Cahill DP, Iafrate AJ, Aldape K, Nutt CL, Louis DN (2009). MSH6 mutations arise in glioblastomas during temozolomide therapy and mediate temozolomide resistance. Clin Cancer Res.

[55] Cahill DP, Levine KK, Betensky RA, Codd PJ, Romany CA, Reavie LB, Batchelor TT, Futreal PA, Stratton MR, Curry WT, Iafrate AJ, Louis DN (2007). Loss of the mismatch repair protein MSH6 in human glioblastomas is associated with tumor progression during temozolomide treatment. Clin Cancer Res.

[56] Hunter C, Smith R, Cahill DP (2006). A hypermutation phenotype and somatic MSH6 mutations in human malignant gliomas after alkylate or chemotherapy. Cancer Res.

[57] Roos WP, Batista LF, Naumann SC, Wick W, Weller M, Menck CF, Kaina B (2007). Apoptosis in malignant glioma cells triggered by the temozolomide induced DNA lesion O6-methylguanine. Oncogene.

[58] Sarkaria JN, Kitange GJ, James CD, Plummer R, Calvert H, Weller M, Wick W (2008). Mechanisms of Chemoresistance to Alkylating Agents in Malignant Glioma. Clinical Cancer Research.

[59] Shen L, Kondo Y, Rosner GL (2005). MGMT promoter methylation and field defect in sporadic colorectal cancer. J Natl Cancer Inst.

[60] Esteller M, Toyota M, Sanchez-Cespedes M (2000). Inactivation of the DNA repair gene O6-methylguanine-DNA methyltransferase by promoter hypermethylation is associated with G to A mutations in K-ras in colorectal tumorigenesis. Cancer Res.

[61] Pegg AE (2000). Repair of O(6)-alkylguanine by alkyltransferases. Mutat Res.

[62] Kaina B, Ochs K, Grosch S (2001). BER, MGMT, and MMR in defense against alkylation-induced genotoxicity and apoptosis. Prog Nucleic Acid Res Mol Biol.

[63] Qian X, von Wronski MA, Brent TP (1995). Localization of methylation sites in the human O6-methylguanine-DNA methyltransferase promoter: correlation with gene suppression. Carcinogenesis.

[64] Esteller M, Herman JG (2004). Generating mutations but providing chemosensitivity: the role of O6-methylguanine DNA methyltransferase in human cancer. Oncogene.

[65] Pietrantonio F, Perrone F, de Braud F, Castano A, Maggi C, Bossi I, Gevorgyan A, Biondani P, Pacifici M, Busico A, Gariboldi M, Festinese F, Tamborini E, Di Bartolomeo M (2014). Activity of temozolomide in patients with advanced chemorefractory colorectal cancer and MGMT promoter methylation. Ann Oncol.

[66] Caraglia M, Addeo R, Costanzo R, Montella L, Faiola V, Marra M, Abbruzzese A, Palmieri G, Budillon A, Grillone F, Venuta S, Tagliaferri P, Del Prete S (2006). Phase II study of temozolomide plus pegylated liposomal doxorubicin in the treatment of brain metastases from solid tumours. Cancer Chemother Pharmacol.

[67] Gonzalez Z, Carlsen L, El-Deiry WS (2023). Temozolomide combined with ipilimumab plus nivolumab enhances T cell killing of MGMT-expressing, MSS colorectal cancer cells. Am J Cancer Res.

[68] Morano F, Raimondi A, Pagani F, Lonardi S, Salvatore L, Cremolini C (2022). Temozolomide followed by combination with low-dose ipilimumaband nivolumab in patients with microsatellite-stable, O6-methylguanine-DNA methyltransferase-silenced metastatic colorectal cancer: the MAYA trial. J Clin Oncol.

[69] Crisafulli G, Sartore-Bianchi A, Lazzari L, Pietrantonio F, Amatu A, Macagno M (2022). Temozolomide treatment alters mismatch repair and boosts mutational burden in tumor and blood of colorectal cancer patients. Cancer Discov.

[70] Stupp R, Mason WP, van den Bent MJ, Weller M, Fisher B, Taphoorn MJ (2005). Radiotherapy plus concomitant and adjuvant temozolomide for glioblastoma. N Engl J Med.

[71] Kulke MH, Stuart K, Enzinger PC, Ryan DP, Clark JW, Muzikansky A (2006). Phase II study of temozolomide and thalidomide in patients with metastatic neuroendocrine tumors. J Clin Oncol.

[72] Ekeblad S, Sundin A, Janson ET, Welin S, Granberg D, Kindmark H (2007). Temozolomideas monotherapy is effective in treatment of advanced malignant neuroendocrine tumors. Clin Cancer Res.

[73] Maire F, Hammel P, Faivre S, Hentic O, Yapur L, Larroque B, Couvelard A, Zappa M, Raymond E, Lévy P, Ruszniewski P (2009). Temozolomide: a safe and effective treatment for malignant digestive endocrine tumors. Neuroendocrinology.

[74] Isacoff WH, Moss RA, Pecora AL, Fine RL (2006). Temozolomide/capecitabine therapy for metastatic neuroendocrine tumors of the pancreas. A retrospective review. J Clin Oncol.

[75] Kulke MH (2006). A phase II study of temozolomide and bevacizumab in patients with advanced neuroendocrine tumors. J Clin Oncol.

[76] Kulke M, Frauenhoffer C, Hooshmand D, Ryan P (2007). Prediction of response to temozolamide (TMZ)-based therapy by loss of MGMT expression in patients with advanced neuroendocrine tumors (NET). In: Proceedings ASCO; 2007; Chicago, IL.

[77] Hirohata T1, Asano K, Ogawa Y, Takano S, Amano K, Isozaki O, Iwai Y, Sakata K, Fukuhara N, Nishioka H, Yamada S, Fujio S, Arita K, Takano K, Tominaga A, Hizuka N, Ikeda H, Osamura RY, Tahara S, Ishii Y, Kawamata T, Shimatsu A, Teramoto A, Matsuno A (2013). DNA mismatch repair protein (MSH6) correlated with the responses of atypical pituitary adenomas and pituitary carcinomas to temozolomide: the national cooperative study by the Japan Society for Hypothalamic and Pituitary Tumors. J Clin Endocrinol Metab.

[78] Sahu A, Jefford M, Lai-Kwon J, Thai A, Hicks RJ, Michael M (2019). CAPTEM in Metastatic Well-Differentiated Intermediate to High Grade Neuroendocrine Tumors: a Single Centre Experience. J Oncol.

[79] Thomas K, Voros BA, Meadows-Taylor M (2020). Outcomes of Capecitabine and Temozolomide (CAPTEM) in Advanced Neuroendocrine Neoplasms (NENs). Cancers.

[80] Jeong H, Shin J, Jeong JH (2021). Capecitabine plus temozolomide in patients with grade 3 unresectable or metastatic gastroenteropancreatic neuroendocrine neoplasms with Ki-67 index <55%: single-arm phase II study. ESMO Open.

[81] Kunz P, Catalano P, Nimeiri H (2018). A randomized study of temozolomide or temozolomide and capecitabine in patients with advanced pancreatic neuroendocrine tumors: a trial of the ECOG-ACRIN Cancer Research Group (E2211). J Clin Oncol.

[82] Peixoto RD, Noonan KL, Pavlovich P, Kennecke HF, Lim HJ (2014). Outcomes of patients treated with capecitabine and temozolamide for advanced pancreatic neuroendocrine tumors (PNETs) and non-PNETs. J Gastrointest Oncol.

[83] Yao JC, Fazio N, Singh S (2016). Everolimus for the treatment of advanced, non-functional neuroendocrine tumours of the lung or gastrointestinal tract (RADIANT-4): a randomised, placebo-controlled, phase 3 study. Lancet.

[84] Wiedmann MW, Mössner J (2012). Safety and efficacy of sunitinib in patients with unresectable pancreatic neuroendocrine tumors. Clin Med Insights Oncol.

[85] Arrivi G, Verrico M, Roberto M, Barchiesi G, Faggiano A, Marchetti P, Mazzuca F, Tomao S (2022). Capecitabine and Temozolomide (CAPTEM) in Advanced Neuroendocrine Neoplasms (NENs): A Systematic Review and Pooled Analysis. Cancer Manag Res.

[86] Canadian Pharmacists Association (2005). Compendium of Pharmaceuticals and Specialties 2005. Ottawa: Canadian Pharmacists Association.

[87] Christodoulou C, Bafaloukos D, Linardou H (2005). Temozolomide (TMZ) combined with cisplatin (CDDP) in patients with brain metastases from solid tumours: a Hellenic Cooperative Oncology Group (HeCOG) Phase II study. J Neuro-Onc.

[88] Christodoulou C, Bafaloukos D, Kosmidis P (2001). for the Hellenic Cooperative Oncology Group. Phase II study of temozolomide in heavily pretreated cancer patients with brain metastases. Ann Oncol.

[89] Abrey LE, Olson JD, Raizer JJ (2001). A phase II trial of temozolomide forpatients with recurrent or progressive brain metastases. J. Neuro-Onc.

[90] Trudeau ME, Crump M, Charpentier D, Yelle L, Bordeleau L, Matthews S, Eisenhauer E (2006). Temozolomide in metastatic breast cancer (MBC): a phase II trial of the National Cancer Institute of Canada - Clinical Trials Group (NCIC-CTG). Ann Oncol.

[91] Hoffmann AL, Buhk JH, Strik H (2009). Neoplastic meningitis from breast cancer: feasibility and activity of long-term intrathecal liposomal Ara-C combined with dose-dense temozolomide. Anticancer Res.

[92] Jenkins S, Zhang W, Steinberg SM, Nousome D, Houston N, Wu X, Armstrong TS, Burton E, Smart DD, Shah R, Peer CJ, Mozarsky B, Arisa O, Figg WD, Mendoza TR, Vera E, Brastianos P, Carter S, Gilbert MR, Anders CK, Connolly RM, Tweed C, Smith KL, Khan I, Lipkowitz S, Steeg PS, Zimmer AS Phase I Study, Cell-Free DNA Analysis of T-DM1, Metronomic Temozolomide for Secondary Prevention of HER2-Positive Breast Cancer Brain Metastases Clin Cancer Res. 2023;29(8):1450-1459.

[93] Patel M, McCully C, Godwin K, Balis FM:Plasma, cerebrospinal fluid pharmacokinetics of intravenous temozolomide in nonhuman primates J Neurol Oncol. 2003; 61:203-207.

[94] Adonizio CS, Babb JS, Maiale C, Huang C, Donahue J, Millenson MM, Hosford M, Somer R, Treat J, Sherman E, Langer CJ (2002). Temozolomide in non-small-cell lung cancer: preliminary results of a phase II trial in previously treated patients. Clin Lung Cancer.

[95] Sorensen JB, Hansen HH, Hansen M, Dombernowsky P (1988). Brain metastases in adenocarcinoma of the lung: frequency, risk groups, and prognosis. J Clin Oncol.

[96] Law A, Karp DD, Dipetrillo T, Daly BT Emergence of increased cerebral metastasis after high-dose preoperative radiotherapy with chemotherapy in patients with locally advanced non-small cell lung carcinoma Cancer. 2001; 92: 160-164.

[97] Kouroussis C1, Vamvakas L, Vardakis N, Kotsakis A, Kalykaki A, Kalbakis K, Saridaki Z, Kentepozidis N, Giassas S, Georgoulias V (2009). Continuous administration of daily low-dose temozolomide in pretreated patients with advanced non-small cell lung cancer: a phase II study. Oncology.

[98] Dziadziuszko R, Ardizzoni A, Postmus PE, Smit EF, Price A, Debruyne C, Legrand C, Giaccone G (2003). Temozolomide in patients with advanced non-small cell lung cancer with and without brain metastases, a phase II study of the EORTC Lung Cancer Group (08965). Eur J Cancer.

[99] Green RA, Humphrey E, Close H, Patno ME (1969). Alkylating agents in bronchogenic carcinoma. Am J Med.

[100] Nugent JL, Bunn PA Jr, Matthews MJ, Ihde DC, Cohen MH, Gazdar A (1979). CNS metastases in small cell bronchogenic carcinoma: increasing frequency and changing pattern with lengthening survival. Cancer.

[101] Toyooka S, Toyooka KO, Maruyama R, Virmani AK, Girard L, Miyajima K (2001). DNA methylation profiles of lung tumors. Mol Cancer Ther.

[102] Zauderer M, Krug LM, Pietanza MC, O'Rourke D (2010). Leptomeningeal metastases from small cell lung cancer responsive to temozolomide therapy. J Thorac Oncol.

[103] Pietanza MC, Kadota K, Huberman K, Sima CS, Fiore JJ, Sumner DK, Travis WD, Heguy A, Ginsberg MS, Holodny AI, Chan TA, Rizvi NA, Azzoli CG, Riely GJ, Kris MG, Krug LM Phase II trial of temozolomide in patients with relapsed sensitive or refractory small cell lung cancer, with assessment of methylguanine-DNA methyltransferase as a potential biomarker Clin Cancer Res. 2012 Feb 15;18(4):1138-45.

[104] Deng X, Zheng Z, Lin B, Su H, Chen H, Fei S (2017). The efficacy and roles of combining temozolomide with whole brain radiotherapy in protection neurocognitive function and improvement quality of life of non-small-cell lung cancer patients with brain metastases. BMC Cancer.

[105] Han J, Qiu M, Su L, Wu C, Cheng S, Zhao Z, Li D, Wang M, Tao W, Du S Response, safety of whole-brain radiotherapy plus temozolomide for patients with brain metastases of non-small-cell lung cancer (2021). A meta-analysis. Thorac Cancer.

[106] van Brussel JP, Busstra MB, Lang MS, Catsburg T, Schroder FH, Mickisch GH (2000). A phase II study of temozolomide in hormone-refractory prostate cancer. Cancer Chemother Pharmacol.

[107] Duesberg P, Li R, Sachs R, Fabarius A, Upender MB, Hehlmann R (2007). Cancer drug resistance: the central role of the karyotype. Drug Resist Updat.

[108] Braun K, Ehemann V, Wiessler M, Pipkorn R, Didinger B, Mueller G, Waldeck W (2009). High-resolution flow cytometry: a suitable tool for monitoring aneuploid prostate cancer cells after TMZ and TMZ-BioShuttle treatment. Int J Med Sci.

[109] Pipkorn R, Waldeck W, Didinger B, Koch M, Mueller G, Wiessler M, Braun K (2009). Inverse-electron-demand Diels-Alder reaction as a highly efficient chemoselective ligation procedure: synthesis and function of a BioShuttle for temozolomide transport into prostate cancer cells. J Pept Sci.

[110] Hussain M1, Carducci MA, Slovin S, Cetnar J, Qian J, McKeegan EM, Refici-Buhr M, Chyla B, Shepherd SP, Giranda VL, Alumkal JJ (2014). Targeting DNA repair with combination veliparib (ABT-888) and temozolomide in patients with metastatic castration-resistant prostate cancer. Invest New Drugs.

[111] Houghton PJ, Stewart CF, Cheshire PJ (2000). Antitumor activity of temozolomide combined with irinotecan is partly independent of O6-methylguanine-DNA methyltransferase and mismatch repair phenotypes in xenograft models. Clin Cancer Res.

[112] Middlemas DS, Stewart CF, Kirstein MN (2000). Biochemical correlates of temozolomide sensitivity in pediatric solid tumor xenograft models. Clin Cancer Res.

[113] Aksoy S, Abali H, Kilickap S (2005). Successful treatment of a chemoresistant tumor with temozolomide in an adult patient: report of a recurrent intracranial mesenchymal chondrosarcoma. J Neurooncol.

[114] Anderson PM, Pearson M (2006). Novel therapeutic approaches in pediatric and young adult sarcomas. Curr Oncol Rep.

[115] Wagner LM, McAllister N, Goldsby RE (2007). Temozolomide and intravenous irinotecan for treatment of advanced Ewing sarcoma. Pediatr Blood Cancer.

[116] Wagner LM, Crews KR, Iacono LC (2004). Phase I trial of temozolomide and protracted irinotecan in pediatric patients with refractory solid tumors. Clin Cancer Res.

[117] Kushner BH, Kramer K, Modak S (2006). Irinotecan plus temozolomide for relapsed or refractory neuroblastoma. J Clin Oncol.

[118] De Angulo G, Hernandez M, Morales-Arias J (2007). Early lymphocyte recovery as a prognostic indicator for high-risk Ewing sarcoma. J Pediatr Hematol Oncol.

[119] Losa R, Fra J, Lopez-Pousa A (2007). Phase II study with the combination of gemcitabine and DTIC in patients with advanced soft tissue sarcomas. Cancer Chemother Pharmacol.

[120] Awada A, Gil T, Sales F (2004). Prolonged schedule of temozolomide (Temodal) plus liposomal doxorubicin (Caelyx) in advanced solid cancers. Anticancer Drugs.

[121] Talbot SM, Keohan ML, Hesdorffer M, Orrico R, Bagiella E, Troxel AB (2003). A phase II trial of temozolomide in patients with unresectable or metastatic soft tissue sarcoma. Cancer.

[122] Trent JC, Beach J, Burgess MA, Papadopolous N, Chen LL, Benjamin RS (2003). A two-arm phase II study of temozolomide in patients with advanced gastrointestinal stromal tumors and other soft tissue sarcomas. Cancer.

[123] Noh JJ, Cho YJ, Ryu JY, Choi JJ, Hwang JR, Choi JY, Lee JW Anticancer Activity of the Combination of Cabozantinib, Temozolomide in Uterine Sarcoma Clin Cancer Res. 2022;28(17):3850-3861.

[124] Nicholson HS, Krailo M, Ames MM (1998). Phase I study of temozolomide in children and adolescents with recurrent solid tumors: A report from the Children's Cancer Group. J Clin Oncol.

[125] Lashford LS, Thiesse P, Jouvet A (2002). Temozolomide in malignant gliomas in childhood: A United Kingdom Children's Cancer Study Group and French Society for Pediatric Oncology Intergroup Study. J Clin Oncol.

[126] De Sio L, Milano GM, Castellano A, Jenkner A, Fidani P, Dominici C, Donfrancesco A (2006). Temozolomide in resistant or relapsed pediatric solid tumors. Pediatr Blood Cancer.

[127] Geoerger B, Vassal G, Doz F, O'Quigley J, Wartelle M, Watson AJ, Raquin MA, Frappaz D, Chastagner P, Gentet JC, Rubie H, Couanet D, Geoffray A, Djafari L, Margison GP, Pein F (2005). Dose finding and O6-alkylguanine-DNA alkyltransferase study of cisplatin combined withtemozolomide in paediatric solid malignancies. Br J Cancer.

[128] Jakacki RI, Hamilton M, Gilbertson RJ, Blaney SM, Tersak J, Krailo MD, Ingle AM, Voss SD, Dancey JE, Adamson PC (2008). Pediatric phase I and pharmacokinetic study of erlotinib followed by the combination of erlotinib and temozolomide: a Children's Oncology Group Phase I Consortium Study. J Clin Oncol.

[129] Casey DA, Wexler LH, Merchant MS (2009). Irinotecan and temozolomide for Ewing sarcoma: The Memorial Sloan-Kettering experience. Pediatr Blood Cancer.

[130] Anwar MA (2024). Blood-Based Multiomics-Guided Detection of a Precancerous Pancreatic Tumor. OMICS.

[131] Bagatell R, London WB, Wagner LM (2011). Phase II study of irinotecan and temozolomide in children with relapsed or refractory neuroblastoma: A Children's Oncology Group study. J Clin Oncol.

[132] Wagner L, Turpin B, Nagarajan R, Weiss B, Cripe T, Geller JPilot study of vincristine, oral irinotecan, temozolomide (VOIT regimen) combined with bevacizumab in pediatric patients with recurrent solid tumors or brain tumors Pediatr Blood Cancer. 2013;60(9):1447-51.

[133] Hadoux J, Favier J, Scoazec JY, Leboulleux S, Al GA, Caramella C (2014). SDHB mutations are associated with response to temozolomide in patients with metastatic pheochromocytoma or paraganglioma. Int J Cancer.

[134] Perez K, Perez K, Jacene H, Hornick JL, Ma C, Vaz N (2022). SDHx mutations and temozolomide in malignant pheochromocytoma and paraganglioma. Endocr Relat Cancer.

[135] Tong A, Li M, Cui Y, Ma X, Wang H, Li Y (2020). Temozolomide is a potential therapeutic tool for patients with metastatic Pheochromocytoma/Paraganglioma- case report and review of the literature. Front Endocrinol (Lausanne).

[136] Geng X, Zhang Y, Lin X, Zeng Z, Hu J, Hao L (2022). Exosomal circWDR62 promotes temozolomide resistance and malignant progression through regulation of the miR-370-3p/MGMT axis in glioma. Cell Death & Disease.

[137] Zhou Y, Chen L, Ding D, Li Z, Cheng L, You Q (2022). Cyanidin-3-O-glucoside inhibits the β-catenin/MGMT pathway by upregulatingmiR-214-5p to reverse chemotherapy resistance in glioma cells. Scientific Reports.

[138] Cheng M, Wang Q, Chen L, Zhao D, Tang J, Xu J (2022). LncRNA UCA1/miR-182-5p/MGMT axis modulates glioma cell sensitivity to temozolomide through MGMT-related DNA damage pathways. Human Pathology.

[139] Li Q, Ren B, Gui Q, Zhao J, Wu M, Shen M (2020). Blocking MAPK/ERK pathway sensitizes hepatocellular carcinoma cells to temozolomide via downregulating MGMT expression. Annals of Translational Medicine.

[140] Jin L, Kiang KMY, Cheng SY, Leung GKK (2022). Pharmacological inhibition of serine synthesis enhances temozolomide efficacy by decreasing O6-methylguanine DNA methyltransferase (MGMT) expression and reactive oxygen species (ROS)-mediated DNA damage in glioblastoma. Laboratory Investigation.

[141] Tang Q, Cao H, Tong N, Liu Y, Wang W, Zou Y (2022). Tubeimoside-I sensitizes temozolomide-resistant glioblastoma cells to chemotherapy by reducing MGMT expression and suppressing EGFR induced PI3K/Akt/mTOR/NF-κB-mediated signaling pathway. Phytomedicine.

[142] Liu Y, Du Z, Xu Z, Jin T, Xu K, Huang M (2021). Overexpressed GNA13 induces temozolomide sensitization via downregulating MGMT and p-RELA in glioma. American Journal of Translational Research.

[143] Li H, Liu S, Jin R, Xu H, Li Y, Chen Y (2021). Pyrvinium pamoate regulates MGMT expression through suppressing the Wnt/β-catenin signaling pathway to enhance the glioblastoma sensitivity to temozolomide. Cell Death Discovery.

[144] Shi J, Chen G, Dong X, Li H, Li S, Cheng S (2021). METTL3 Promotes the Resistance of Glioma to Temozolomide via Increasing MGMT and ANPG in a m6A Dependent Manner. Frontiers in Oncology.

[145] Lv W, Li Q, Jia B, He Y, Ru Y, Guo Q (2021). Differentiated embryonic chondrocyte-expressed gene 1 promotes temozolomide resistance by modulating the SP1-MGMT axis in glioblastoma. American Journal of Translational Research.

[146] Lin K, Gueble SE, Sundaram RK, Huseman ED, Bindra RS, Herzon SB (2022). Mechanism-based design of agents that selectively target drug-resistant glioma. Science.

[147] Delaney CA, Wang LZ, Kyle S, White AW, Calvert AH, Curtin NJ (2000). Potentiation of temozolomide and topotecan growth inhibition and cytotoxicity by novel poly(adenosine diphosphoribose) polymerase inhibitors in a panel of human tumor cell lines. Clin Cancer Res.

[148] Murai J, Huang SYN, Das BB, Renaud A, Zhang Y, Doroshow JH (2012). Trapping of PARP1 and PARP2 by clinical PARP inhibitors. Cancer Res.

[149] Murai J, Zhang Y, Morris J, Ji J, Takeda S, Doroshow JH (2014). Rationale for Poly(ADP-ribose) polymerase (PARP) inhibitors in combination therapy with camptothecins or temozolomide based on parp trapping versus catalytic inhibition. J Pharmacol Exp Ther.

[150] Pommier Y, O'Connor MJ, de Bono J (2016). Laying a trap to kill cancer cells: PARP inhibitors and their mechanisms of action. Sci Transl Med.

[151] Hopkins TA, Ainsworth WB, Ellis PA, Donawho CK, DiGiammarino EL, Panchal SC (2019). PARP1 trapping by PARP inhibitors drives cytotoxicity in both cancercells and healthy bone marrow. Mol Cancer Res.

[152] Cecchini M, Zhang JY, Wei W, Sklar J, Lacy J, Zhong M, Kong Y, Zhao H, DiPalermo J, Devine L, Stein SM, Kortmansky J, Johung KL, Bindra RS, LoRusso P, Schalper KA Quantitative DNA Repair Biomarkers, Immune Profiling for Temozolomide, Olaparib in Metastatic Colorectal Cancer Cancer Res Commun. 2023;3(6):1132-1139.

[153] Johannessen TC, Bjerkvig R.Molecular mechanisms of temozolomide resistance in glioblastoma multiforme Expert Rev Anticancer Ther. 2012;12: 635-42.

[154] Lee SY (2016). Temozolomide resistance in glioblastoma multiforme. Genes Dis.

[155] Dexheimer TS, Coussens NP, Silvers T, Wright J, Morris J, Doroshow JH, Teicher BA Multicellular Complex Tumor Spheroid Response to DNA Repair Inhibitors in Combination with DNA-damaging Drugs Cancer Res Commun. 2023;3(8):1648-1661.

[156] Tentori L, Ricci-Vitiani L, Muzi A, Ciccarone F, Pelacchi F, Calabrese R (2014). Pharmacological inhibition of poly(ADP-ribose) polymerase-1 modulates resistance of human glioblastoma stem cells to temozolomide. BMC Cancer.

[157] Roos WP, Kaina B (2013). DNA damage-induced cell death: from specific DNA lesions to the DNA damage response and apoptosis. Cancer Lett.

[158] Wu S, Li X, Gao F, de Groot JF, Koul D, Yung WKA (2021). PARP-mediated PARylation of MGMT is critical to promote repair of temozolomide-induced O6-methylguanine DNA damage in glioblastoma. Neuro Oncol.

[159] Smith MA, Reynolds CP, Kang MH, Kolb EA, Gorlick R, Carol H (2015). Synergistic activity of PARP inhibition by talazoparib (BMN 673) with temozolomide in pediatric cancer models in the pediatric preclinical testing program. Clin Cancer Res.

[160] Ueno D, Vasquez JC, Sule A, Liang J, van Doorn J, Sundaram R (2022). Targeting Krebs-cycle-deficient renal cell carcinoma with Poly ADP-ribose polymerase inhibitors and low-dose alkylating chemotherapy. Oncotarget.

[161] Lok BH, Gardner EE, Schneeberger VE, Ni A, Desmeules P, Rekhtman N (2017). PARP inhibitor activity correlates with SLFN11 expression and demonstrates synergy with temozolomide in small cell lung cancer. Clin Cancer Res.

[162] Karachi A, Dastmalchi F, Mitchell DA, Rahman M (2018). Temozolomide for immunomodulation in the treatment of glioblastoma. Neuro Oncol.

[163] Kim TG, Kim CH, Park JS, Park SD, Kim CK, Chung DS, Hong YK (2010). Immunological factors relating to the antitumor effect of temozolomide chemoimmunotherapy in a murine glioma model. Clin Vaccine Immunol.

[164] Weiss T, Weller M, Roth P (2016). Immunological effects of chemotherapy and radiotherapy against brain tumors. Expert Review of Anticancer Therapy.

[165] Hotchkiss KM, Sampson JH (2021). Temozolomide treatment outcomes and immunotherapy efficacy in brain tumor. J Neurooncol.

[166] Natalia Di Ianni, Martina Maffezzini, Marica Eoli, Serena Pellegatta (2021). Revisiting the Immunological Aspects of Temozolomide Considering the Genetic Landscape and the Immune Microenvironment Composition of Glioblastoma. Front Oncol.

[167] Stupp R, Hegi ME, Mason WP (2009). Effects of radiotherapy with concomitant and adjuvant temozolomide versus radiotherapy alone on survival in glioblastoma in a randomized phase III study: 5-year analysis of the EORTC-NCIC trial. Lancet Oncol.

[168] Grossman SA (2017). A search for the “Goldilocks zone” with regard to the optimal duration of adjuvant temozolomide in patients with glioblastoma. Neuro-Oncol.

[169] Balana C (2022). Optimal duration of adjuvant temozolomide in glioblastoma: unsolved and unsolvable problem. Neurooncol Pract.

[170] Nabors LB, Lamb LS, Goswami T, Rochlin K, Youngblood SL (2024). Adoptive cell therapy for high grade gliomas using simultaneous temozolomide and intracranial mgmt-modified γδ t cells following standard post-resection chemotherapy and radiotherapy: current strategy and future directions. Immunol.

